# Effect of Early vs Late Supplemental Parenteral Nutrition in Patients Undergoing Abdominal Surgery: A Randomized Clinical Trial

**DOI:** 10.1001/jamasurg.2022.0269

**Published:** 2022-03-16

**Authors:** Xuejin Gao, Yuxiu Liu, Li Zhang, Da Zhou, Feng Tian, Tingting Gao, Hao Tian, Hao Hu, Fangyou Gong, Dong Guo, Junde Zhou, Yingchao Gu, Bo Lian, Zhigang Xue, Zhenyi Jia, Zhida Chen, Yong Wang, Gang Jin, Kunhua Wang, Yanbing Zhou, Qiang Chi, Hua Yang, Mengbin Li, Jianchun Yu, Huanlong Qin, Yun Tang, Xiaoting Wu, Guoli Li, Ning Li, Jieshou Li, Claude Pichard, Xinying Wang

**Affiliations:** 1 Department of General Surgery, Jinling Hospital, Medical School of Nanjing University, Nanjing, China; 2 Department of Medical Statistics, Jinling Hospital, Nanjing Medical University, Nanjing, China; 3 Department of Biostatistics, School of Public Health, Southern Medical University, Guangzhou, China; 4 Department of Hepatobiliary Pancreatic Surgery, Changhai Hospital, the Second Military Medical University, Shanghai, China; 5 Department of General Surgery, First Affiliated Hospital of Kunming Medical University, Kunming, China; 6 Department of Gastrointestinal Surgery, the Affiliated Hospital of Qingdao University, Qingdao, China; 7 Department of General Surgery, the Second Affiliated Hospital of Harbin Medical University, Harbin, China; 8 Department of General Surgery, the Second Affiliated Hospital, Army Medical University, Chongqing, China; 9 Department of Gastrointestinal Surgery, the First Affiliated Hospital of Air Force Medical University, Xi’an, China; 10 Department of General Surgery, Peking Union Medical College Hospital, Chinese Academy of Medical Sciences, Beijing, China; 11 Department of General Surgery, Shanghai Tenth People's Hospital, School of Medicine, Tongji University, Shanghai, China; 12 Department of General Surgery, First Medical Center of Chinese PLA General Hospital, Beijing, China; 13 Department of Gastrointestinal Surgery, West China Hospital, Sichuan University, Chengdu, China; 14 Nutrition Unit, Geneva University Hospital, Lausanne, Switzerland

## Abstract

**Importance:**

The effect of and optimal timing for initiating supplemental parenteral nutrition (SPN) remain unclear after major abdominal surgery for patients in whom energy targets cannot be met by enteral nutrition (EN) alone.

**Objective:**

To examine the effect of early supplemental parenteral nutrition (E-SPN) (day 3 after surgery) or late supplemental parenteral nutrition (L-SPN) (day 8 after surgery) on the incidence of nosocomial infections in patients undergoing major abdominal surgery who are at high nutritional risk and have poor tolerance to EN.

**Design, Setting, and Participants:**

A multicenter randomized clinical trial was conducted from April 1, 2017, to December 31, 2018, in the general surgery department of 11 tertiary hospitals in China. Participants were those undergoing major abdominal surgery with high nutritional risk and poor tolerance to EN (≤30% of energy targets from EN on postoperative day 2, calculated as 25 and 30 kcal/kg of ideal body weight daily for women and men, respectively) and an expected postoperative hospital stay longer than 7 days. Data analysis was performed from February 1 to October 31, 2020.

**Interventions:**

Random allocation to E-SPN (starting on day 3 after surgery) or L-SPN (starting on day 8 after surgery).

**Main Outcomes and Measures:**

The primary outcome was the incidence of nosocomial infections between postoperative day 3 and hospital discharge.

**Results:**

A total of 230 patients (mean [SD] age, 60.1 [11.2] years; 140 men [61.1%]; all patients were of Han race and Asian ethnicity) were randomized (115 to the E-SPN group and 115 to the L-SPN group). One patient in the L-SPN group withdrew informed consent before the intervention. The E-SPN group received more mean (SD) energy delivery between days 3 and 7 compared with the L-SPN group (26.5 [7.4] vs 15.1 [4.8] kcal/kg daily; *P* < .001). The E-SPN group had significantly fewer nosocomial infections compared with the L-SPN group (10/115 [8.7%] vs 21/114 [18.4%]; risk difference, 9.7%; 95% CI, 0.9%-18.5%; *P* = .04). No significant differences were found between the E-SPN group and the L-SPN group in the mean (SD) number of noninfectious complications (31/115 [27.0%] vs 38/114 [33.3%]; risk difference, 6.4%; 95% CI, −5.5% to 18.2%; *P* = .32), total adverse events (75/115 [65.2%] vs 82/114 [71.9%]; risk difference, 6.7%; 95% CI, −5.3% to 18.7%; *P* = .32), and rates of other secondary outcomes. A significant difference was found in the mean (SD) number of therapeutic antibiotic days between the E-SPN group and the L-SPN group (6.0 [0.8] vs 7.0 [1.1] days; mean difference, 1.0 days; 95% CI, 0.2-1.9 days; *P* = .01).

**Conclusion and Relevance:**

In this randomized clinical trial, E-SPN was associated with reduced nosocomial infections in patients undergoing abdominal surgery and seems to be a favorable strategy for patients with high nutritional risk and poor tolerance to EN after major abdominal surgery.

**Trial Registration:**

ClinicalTrials.gov Identifier: NCT03115957

## Key Points

**Question:**

When should supplemental parenteral nutrition (SPN) after major abdominal surgery be considered for patients in whom energy targets cannot be met by enteral nutrition alone?

**Findings:**

This multicenter randomized clinical trial compared the effect of early supplemental parenteral nutrition (E-SPN) and late supplemental parenteral nutrition (L-SPN) in 230 patients with high nutritional risk and poor tolerance to enteral nutrition after major abdominal surgery. Results showed that E-SPN in combination with enteral nutrition was associated with a reduced incidence of nosocomial infection compared with L-SPN.

**Meaning:**

These findings provide evidence that E-SPN in combination with enteral nutrition after major abdominal surgery is preferable to L-SPN to reduce nosocomial infections.

## Teaser

This randomized clinical trial examines the effect of early vs late supplemental parenteral nutrition on the incidence of nosocomial infections in patients undergoing major abdominal surgery.

## Introduction

The estimated prevalence of malnutrition in patients after major abdominal surgery ranges from 20% to 70%^1,2^ and is associated with increased morbidity, such as impaired wound healing, hospital-acquired infection, postoperative complications, prolonged hospital stay, and increased mortality.^3,4,5^ It is well documented that the catabolic response to surgery causes the depletion of essential nutrients, resulting in an increased risk of postoperative complications, particularly infectious complications. Therefore, timely and adequate energy supply is essential for maintaining optimal cell and organ function, promoting wound repair, and decreasing infectious complications after surgery.

The European Society for Parenteral and Enteral Nutrition (ESPEN) and the Enhanced Recovery After Surgery (ERAS) Society guidelines^2,6,7,8,9^ recommend that enteral nutrition (EN) should be implemented for patients after surgery as soon as possible if the gastrointestinal tract works. Compared with parenteral nutrition (PN), a meta-analysis and several randomized clinical trials^10,11,12,13^ reported that EN is associated with lower postoperative infections, mortality, and length of stay in patients undergoing major abdominal surgery. However, in many cases, energy delivery in postsurgical patients using EN alone is less than the estimated requirements for various reasons. To supplement insufficient EN, PN is a strategy that can increase energy delivery more closely to the estimated energy requirements. However, recommendations for its use differ, and the evidence is controversial.^2,14,15,16,17,18,19^ Current clinical guidelines for PN support in surgical patients are largely based on expert opinion and differ substantially across continents.^2,14,20^ The ESPEN guidelines recommend that surgeons consider initiating PN if the energy requirements (<50% of energy requirement) of the patient have not been met by EN for more than 7 days.^2^ The American Society for Parenteral and Enteral Nutrition guidelines recommend that PN should be initiated within 3 to 5 days for patients who are at nutritional risk and unlikely to achieve a desired oral intake or with insufficient EN (<60% of energy requirement).^14,21,22^

Infectious risk related to PN has been a concern when compared with EN. However, this concern has been challenged in recent trials that investigated PN in critically ill patients^23,24^ or those undergoing abdominal surgery.^25^ One randomized trial^17^ found that early supplemental PN in critically ill patients with insufficient EN can significantly reduce nosocomial infections, and another^18^ found a trend to reduce newly acquired infections in nutritionally at-risk, critically ill patients. Many observational studies^26,27,28^ have suggested an association between higher energy delivery and improved clinical outcomes in critically ill patients. However, there is still a lack of large randomized clinical trials on the timing of supplemental parenteral nutrition (SPN) initiation for patients undergoing abdominal surgery. The objective of this randomized clinical trial was to evaluate the effects of initiating early SPN (E-SPN) (day 3 after surgery) or late SPN (L-SPN) (day 8 after surgery) on the incidence of nosocomial infections in patients undergoing major abdominal surgery who were at nutritional risk and intolerant to EN.

## Methods

### Study Design and Participants

This investigator-initiated, multicenter, open-label randomized clinical trial on nutritional intervention was conducted in the general surgery departments of 11 tertiary hospitals in China from April 1, 2017, to December 31, 2018. A total of 1560 patients were screened. Data analysis was performed from February 1 to October 31, 2020. The trial protocol and the statistical analysis plan are available in Supplement 1. The trial protocol was approved by the Jinling Hospital Ethics Committee and was registered at ClinicalTrials.gov. All participating patients provided written informed consent. This study followed the Consolidated Standards of Reporting Trials (CONSORT) reporting guideline.^29^

The inclusion criteria were as follows: adults patients who underwent elective gastric, colorectal, hepatic, and pancreatic resections (both benign and malignant disease) without traumatic reasons; were at risk of malnutrition defined as a Nutritional Risk Screening 2002 (NRS-2002) score of 3 or higher^30^; were expected to have a postoperative hospital stay longer than 7 days; and had received 30% or less of the energy target by EN on day 2 after surgery (eAppendix in Supplement 2). Detailed exclusion criteria are described in the trial protocol.

### Randomization and Masking

Randomization was performed using a permuted block design, with stratification of different clinical centers (eTable 1 in Supplement 2). The random allocation sequences were computer generated. Allocation concealment was implemented by sequentially numbered, sealed, opaque envelopes. After being deemed eligible for enrollment, patients were randomized in a 1:1 ratio to the E-SPN group or the L-SPN group. Investigators and participants were not masked to the treatment assignment, but the follow-up assessments were performed by trained physicians and nurses who were blinded to the patient’s assignment. The statisticians were blinded to the treatment group during the data analysis.

### Screening and Baseline Measurements

Patients’ preoperative baseline characteristics, including sex, age, weight, height, body mass index, NRS-2002 score, comorbidities, disease diagnosis, and type of tumor (if applicable) were collected. The duration of surgery, operative blood loss, operative characteristics, and the amount of homologous blood transfusions were recorded. Furthermore, preoperative baseline levels of C-reactive protein, white blood cells, albumin, and prealbumin as well as hepatic and kidney function were measured by laboratory testing.

### Procedures

Enteral nutrition was started within 24 hours after abdominal surgery according to standard procedures based on ESPEN guidelines.^2^ Energy targets were calculated as 30 kcal/kg of ideal body weight for men and 25 kcal/kg of ideal body weight for women, and the protein requirements were 1.2 g/kg of ideal body weight.

A trained clinician developed personalized nutritional plans to reach the energy target. These plans were initially based on EN supplements. After the randomization, both groups received nutrition support for a minimum of 5 days, until 80% of the energy target had been reached via EN, or until hospital discharge. Enteral nutrition products were routinely prescribed at all hospitals and contained 1 kcal/mL of energy (16% proteins, 35% lipids, and 49% carbohydrates). Enteral nutrition was performed by tube feeding. Parenteral nutrition formulas consisted of 0.88 kcal/mL of energy (15% proteins, 40% lipids [20% long-chain triglycerides], and 45% carbohydrates) and supplemental vitamins and minerals. Parenteral nutrition was administered via peripheral or central veins.

Eligible patients were randomly assigned to the E-SPN group or the L-SPN group (eFigure 1 in Supplement 2). For patients in the E-SPN group, SPN was initiated on day 3 after surgery to reach the energy target, whereas SPN was initiated on day 8 after surgery for patients in the L-SPN group. The energy target of combined EN and SPN was 100% of the energy requirement. When enteral feeding comprised 80% of the energy goal, SPN was reduced and eventually discontinued.

The energy target in both groups was verified every 24 hours throughout the study period by a trained clinician based on the daily nutritional information records. Daily nutritional information was recorded for a maximum of 12 days or until patients could resume a normal oral diet or discharge. The daily and cumulative energy postoperative results from nutritional products and nonnutritional fluids (eg, glucose for drug dilution and lipids from propofol) were also recorded. We routinely performed blood glucose monitoring on each patient during the hospital stay, especially at SPN initiation.

The patients were monitored for postoperative complications by trained experienced physicians not associated with the surgical teams. According to previously described criteria, complications were classified as major or minor and infectious or noninfectious (eTable 2 in Supplement 2).^13,31^

### Outcomes

The primary outcome was the occurrence of nosocomial infections between postoperative day 3 and discharge. The following infections were defined according to the Centers for Disease Control and Prevention^32^: bloodstream infections, pneumonia, urinary tract infections, surgical site infections, abdominal infections, and other infections (eTable 3 in Supplement 2).

The secondary outcomes included the actual energy and protein intake (including EN and PN), postoperative noninfectious complications, incidence of gastrointestinal intolerance, PN-related complications, length of hospital stay, hospitalization expenses, therapeutic antibiotic days (defined as days from postoperative day 3 to discharge during which a patient received at least 1 dose of antibiotics for actual nosocomial infection), prophylactic antibiotic days (defined as days antibiotics were used for prophylaxis [no infection]), mechanical ventilation, mortality within 2 months after randomization, and laboratory tests at discharge, including white blood cell count, C-reactive protein level, albumin level, prealbumin level, hepatic function, and kidney function.

### Statistical Analysis

A previous systematic meta-analysis study^12^ found an overall infection rate of 10% to 30% in patients after abdominal surgery. That trial assumed an incidence of 25% of nosocomial infections in patients receiving PN after abdominal surgery. We postulated that E-SPN combined with EN might decrease the nosocomial infection rate by 15%. With a 2-tailed type I error rate of 5%, to detect such an effect with a statistical power level of 80%, a sample size of 110 patients would be required in each group. The sample size was increased to 230 to allow for withdrawal and loss to follow-up.

The full analysis set was based on the intention-to-treat principle. Variables are reported as number (percentages), means (SDs), or medians (IQRs) as appropriate. We used the Shapiro-Wilk test to assess whether continuous data were normally distributed. We performed a group comparison with the χ^2^ test or Fisher exact test for categorical variables and the 2-tailed *t* test or Mann-Whitney *U* test for continuous variables when appropriate. The rate of nosocomial infections in a time-to-event analysis was reported using Kaplan-Meier plots, and the difference between the 2 groups was tested by log-rank test. A Cox proportional hazards regression model was used to estimate the hazard ratios and corresponding 95% CIs. We also performed subgroup analyses for the primary outcome, including the following variables: age (<65 vs ≥65 years), sex (male vs female), NRS-2002 score (3 vs ≥4), comorbidity (yes vs no), cancer (yes vs no), operation type (laparotomy vs laparoscope), operation time (≤5 vs >5 hours), and blood loss (≤500 vs >500 mL). No data on primary outcomes were missing. Missing data for the other variables were not imputed. Statistical significance was set as a 2-sided *P* < .05. All analyses were performed using SAS software, version 9.4 (SAS Institute Inc).

## Results

### Study Participants

**Figure 1.**
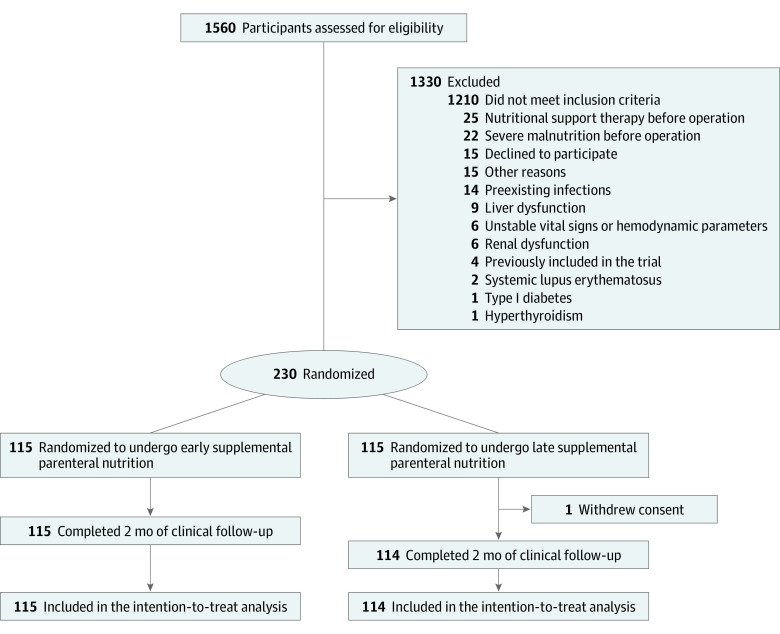
CONSORT Diagram

Of the 1560 screened patients, 230 eligible patients (mean [SD] age, 60.1 [11.2] years; 140 male [61.1%]; all patients were of Han race and Asian ethnicity) were enrolled, with 115 randomized to the E-SPN group and 115 to the L-SPN group. One patient in the L-SPN group withdrew informed consent after randomization and thus did not receive the intervention (Figure 1). At baseline, the characteristics of the patients were similar in the 2 groups (Table 1; eTable 4 in Supplement 2).

**Table 1.** Baseline Demographic and Clinical Characteristics^a^

Characteristic	E-SPN (n = 115)	L-SPN (n = 114)
Sex, No. (%)		
Male	71 (61.7)	69 (60.5)
Female	44 (38.3)	45 (39.5)
Age, y	60.3 (12.2)	59.8 (10.3)
Height, cm	165.1 (8.1)	164.5 (8.4)
Weight, kg	62.7 (11.0)	62.1 (11.4)
BMI	23.0 (3.2)	22.8 (3.0)
NRS-2002 score, No. (%)^b^		
3	92 (80.0)	91 (79.8)
4	21 (18.3)	20 (17.5)
≥5	2 (1.7)	3 (2.6)
Diagnosis, No. (%)		
Gastric cancer	39 (33.9)	36 (31.6)
Colorectal cancer	40 (34.8)	46 (40.4)
Pancreatic cancer	12 (10.4)	17 (14.9)
Cholangiocarcinoma	1 (0.9)	3 (2.6)
Other gastrointestinal cancers	15 (13.0)	8 (7.0)
Benign gastrointestinal disease^c^	8 (7.0)	4 (3.5)
Comorbidity, No. (%)		
Comorbidities^d^	15 (13.0)	15 (13.2)
Nutritional indicators		
Albumin, g/dL	4.11 (0.52)	4.06 (0.46)
Prealbumin, mg/dL	22.40 (6.02)	21.32 (6.05)
Transferrin, mg/dL	236 (66)	210 (74)
Retinol-binding protein, mg/L	37.5 (11.8)	35.0 (11.2)
Hepatic and renal function		
ALT, U/L	24.4 (26.7)	24.6 (20.5)
AST, U/L	24.7 (19.6)	27.6 (22.1)
ALP, U/L	100.4 (107.8)	104.9 (100.0)
Total bilirubin, mg/dL	1.62 (3.82)	1.38 (2.30)
Urea nitrogen, mg/dL	15.27 (5.21)	15.35 (4.62)
Creatinine, mg/dL	0.83 (0.22)	0.81 (0.25)
Metabolism-related index		
Glucose, median (IQR), mg/dL	93.69 (85.77-109.91)	91.89 (84.68-99.10)
Total cholesterol, mg/dL	159.85 (79.54)	160.23 (63.32)
Triglyceride, mg/dL	122.12 (94.69)	134.51 (115.93)
HDL-C, mg/dL	53.67 (38.22)	47.10 (16.22)
LDL-C, mg/dL	100.77 (29.34)	101.54 (31.66)
Inflammatory biomarkers		
White blood cell, /μL	6260 (2370)	5830 (1740)
C-reactive protein, mg/dL	0.93 (2.01)	0.72 (1.27)

Abbreviations: ALP, alkaline phosphatase; ALT, alanine aminotransferase; AST, aspartate aminotransferase; BMI, body mass index (calculated as weight in kilograms divided by height in meters squared); E-SPN, early supplemental parenteral nutrition; HDL-C, high-density lipoprotein cholesterol; LDL-C, low-density lipoprotein cholesterol; L-SPN, late supplemental parenteral nutrition; NRS-2002, Nutritional Risk Screening 2002.

SI conversion factors: To convert albumin to g/L, multiply by 10; prealbumin to mg/L, multiply by 10; transferrin to μmol/L, multiply by 0.123; ALT, AST, and ALP to mckat/L, multiply by 0.0167; total bilirubin to μmol/L, multiply by 17.014; urea nitrogen to mmol/L, multiply by 0.357; creatinine to μmol/L, multiply by 88.4; glucose to mmol/L, multiply by 0.0555; total cholesterol, HDL-C, and LDL-C to mmol/L, multiply by 0.0259; triglycerides to mmol/L, multiply by 0.0113; white blood cells to ×10^9^/L, multiply by 0.001; and C-reactive protein to mg/L, multiply by 10.

^a^ Data are presented as mean (SD) unless otherwise indicated.

^b^ Scores on NRS-2002 range from 0 to 7, with a score of 3 or more identifying patients at nutritional risk. Higher scores indicate an increased risk.

^c^ Diverticular disease, pyloric stenosis, or chronic pancreatitis.

^d^ Comorbidities included type 2 diabetes, urarthritis, and hypertensive diseases.

### Nutrition Therapy

**Figure 2.**
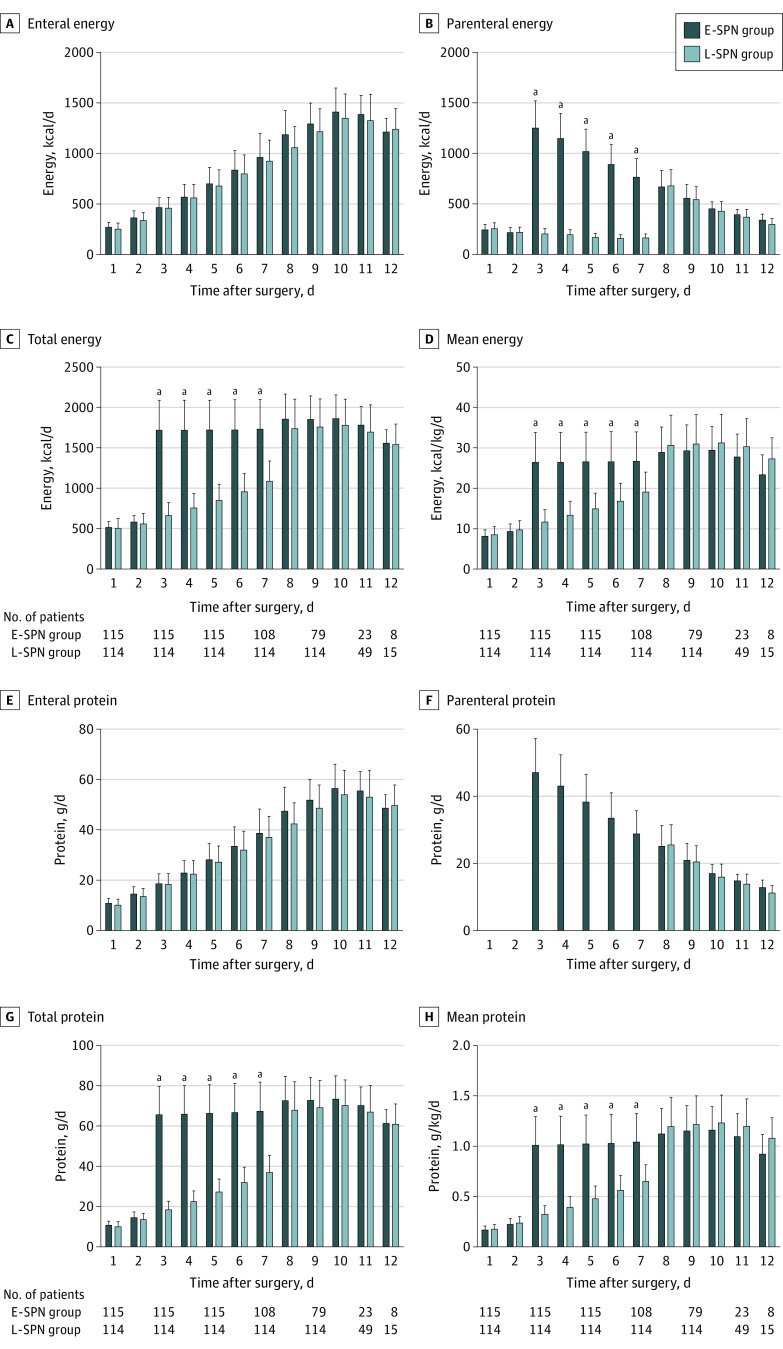
Mean Daily Energy and Protein Intake During the 12 Days After Surgery The daily amount of energy (kilocalories per day) and protein (grams per day) provided by the enteral route, the parenteral route, or both (total) is shown for participants during 12 days after surgery. Error bars indicate SEs. E-SPN indicates early supplemental parenteral nutrition; L-SPN, late supplemental parenteral nutrition. ^a^*P* < .001.

Between days 3 and 7, patients in the E-SPN group received a mean (SD) energy intake of 26.5 (7.4) kcal/kg per day, whereas those in the L-SPN group received a mean (SD) energy intake of 15.1 (4.8) kcal/kg per day (*P* < .001) (Figure 2; eTable 5 in Supplement 2). During the same period, the mean (SD) protein intake was 1.02 (0.28) g/kg per day in the E-SPN group and 0.48 (0.17) g/kg per day in the L-SPN group (*P* < .001 (Figure 2; eTable 6 in Supplement 2). Meanwhile, no statistical differences were found in mean (SD) energy intake (28.8 [6.2] vs 29.6 [7.2] kcal/kg per day; *P* = .17) and mean protein intake (1.17 [0.25] vs 1.20 [0.28] g/kg per day; *P* = .35) between the E-SPN group and the L-SPN group during the 8 to 12 days after surgery (Figure 2; eTable 5 in Supplement 2).

### Primary Clinical Outcome

Overall, the total number of infectious complications in patients in the E-SPN group was significantly less than those in the L-SPN group (10/115 [8.7%] vs 21/114 [18.4%]; risk difference, 9.7%; 95% CI, 0.9%-18.5%; *P* = .04) (Table 2). Kaplan-Meier survival curves plotted with the nosocomial infection rates in the 2 groups also showed a statistically significant difference (hazard ratio, 2.07; 95% CI, 1.01-4.22; *P* = .04) (eFigure 2 in Supplement 2). This significant difference was mainly attributable to the number of major infectious complications, which was significantly lower in the E-SPN group compared with that in the L-SPN group (8/115 [7.0%] vs 18/114 [15.8%]; risk difference, 8.8%; 95% CI, 0.7%-17.0%; *P* = .04) (Table 2). No statistically significant difference was found in the number of minor infectious complications (2/115 [1.7%] vs 3/114 [2.6%]; risk difference, 0.9%; 95% CI, −2.9% to 4.7%; *P* = .68).

**Table 2.** Primary and Secondary Clinical Outcomes During the Intervention and Follow-up^a^

Outcome	E-SPN (n = 115)	L-SPN (n = 114)	Absolute difference (95% CI)	*P* value
Primary outcome				
Infectious complications	10 (8.7)	21 (18.4)	9.7 (0.9 to 18.5)	.04
Major infectious				
Pneumonia	5 (4.3)	11 (9.6)	8.8 (0.7 to 17.0)	.04
Abdominal infection	1 (0.9)	4 (3.5)		
Septic shock	0 (0.0)	2 (1.8)		
Bloodstream infection	2 (1.7)	1 (0.9)		
Minor infectious				
Surgical site infection	1 (0.9)	2 (1.8)	0.9 (−2.9 to 4.7)	.68
Urinary tract infection	1 (0.9)	1 (0.9)		
Secondary outcomes				
Noninfectious complications	31 (27.0)	38 (33.3)	6.4 (−5.5 to 18.2)	.32
Major noninfectious	14 (12.2)	19 (16.7)	4.5 (−4.6 to 13.6)	.35
Minor noninfectious	17 (14.8)	19 (16.7)	1.9 (−7.5 to 11.3)	.72
Total adverse effects	75 (65.2)	82 (71.9)	6.7 (−5.3 to 18.7)	.32
GI intolerance complications	67 (58.3)	79 (69.3)	11.0 (−1.3 to 23.4)	.10
Parenteral nutrition–related complications	9 (7.8)	4 (3.5)	−4.3 (−10.3 to 1.6)	.25
Time in hospital, mean (SD), d	16.6 (8.8)	17.6 (8.4)	1.0 (−1.1 to 3.1)	.39
Mechanical ventilatory support	4 (3.5)	7 (6.1)	2.7 (−2.9 to 8.2)	.38
ICU	7 (6.1)	9 (7.9)	1.8 (−4.8 to 8.4)	.62
Mortality	NA	NA		
Hospitalization costs, mean (SD), ¥^b^	72 959 (30 147)	71 239 (22 942)	−1720 (−8700 to 5260)	.63
Antibiotic days, mean (SD)				
Total	2.9 (1.4)	3.3 (2.0)	0.5 (0.03 to 0.96)	.054
Prophylactic	2.46 (0.74)	2.47 (0.78)	0.01 (−0.20 to 0.23)	.71
Therapeutic	6.0 (0.8)	7.0 (1.1)	1.0 (0.2 to1.9)	.01

Abbreviations: E-SPN, early supplemental parenteral nutrition; GI, gastrointestinal; ICU, intensive care unit; L-SPN, late supplemental parenteral nutrition; NA, not applicable.

^a^ Data are presented as number (percentage) of participants unless otherwise noted. Continuous data, expressed as mean (SD), were compared using the *t* test or Mann-Whitney *U* test. Outcomes expressed as percentages of patients with each outcome were compared between the 2 groups using the Fisher exact test. Parenteral nutrition–related complications were hyperglycemia, hypoglycemia, and hyperlipidemia.

^b^ The current exchange rate of $1 to ¥6.34 was used.

### Secondary Clinical Outcomes

No significant difference was found in the incidence of noninfectious complications between the E-SPN group and the L-SPN group (total noninfectious complications: 31/115 [27.0%] vs 38/114 [33.3%]; risk difference, 6.4%; 95% CI, −5.5% to 18.2%; *P* = .32; major noninfectious complications: 14/115 [12.2%] vs 19/114 [16.7%]; risk difference, 4.5%; 95% CI, −4.6% to 13.6%; *P* = .35; minor noninfectious complications: 17/115 [14.8%] vs 19/114 [16.7%]; risk difference, 1.9%; 95% CI, −7.5% to 11.3%; *P* = .72) (Table 2; eTable 6 in Supplement 2). No significant difference was found in the total incidence of adverse events between the 2 groups (E-SPN vs L-SPN: 75/115 [65.2%] vs 82/114 [71.9%]; risk difference, 6.7%; 95% CI, −5.3% to 18.7%; *P* = .32) (Table 2; eTable 7 in Supplement 2). Patients in the L-SPN group had slightly increased gastrointestinal intolerance events, but this difference was not significant (E-SPN vs L-SPN: 67/115 [58.3%] vs 79/114 [69.3%]; risk difference, 11.0%; 95% CI, −1.3% to 23.4%; *P* = .10) (Table 2; eTable 7 in Supplement 2).

The mean (SD) number of therapeutic antibiotic days was significantly lower in the E-SPN group than in the L-SPN group (6.0 [0.7] vs 7.0 [1.1] days; mean difference, 1.0; 95% CI, 0.2%-1.9%; *P* = .01) (Table 2). No significant differences were found between the 2 groups in any other secondary outcomes.

Mean (SD) serum albumin and prealbumin levels at discharge were significantly higher in the E-SPN group than in the L-SPN group (albumin: 3.55 [0.76] vs 3.37 [0.45] g/dL; mean difference, 0.19 g/dL; 95% CI, 0.03-0.35 g/dL; *P* = .02 [to convert albumin to grams per liter, multiply by 10]; prealbumin: 15.84 [3.81] vs 13.0 [3.63] mg/dL; mean difference, 2.85 mg/dL; 95% CI, 1.88-3.82 mg/dL; *P* < .001 [to convert prealbumin to milligrams per liter, multiply by 10]) (eTable 8 in Supplement 2). No significant differences were found in the rest of the hematologic indicators between the 2 groups (eTable 8 in Supplement 2).

### Subgroup Analyses

**Figure 3.**
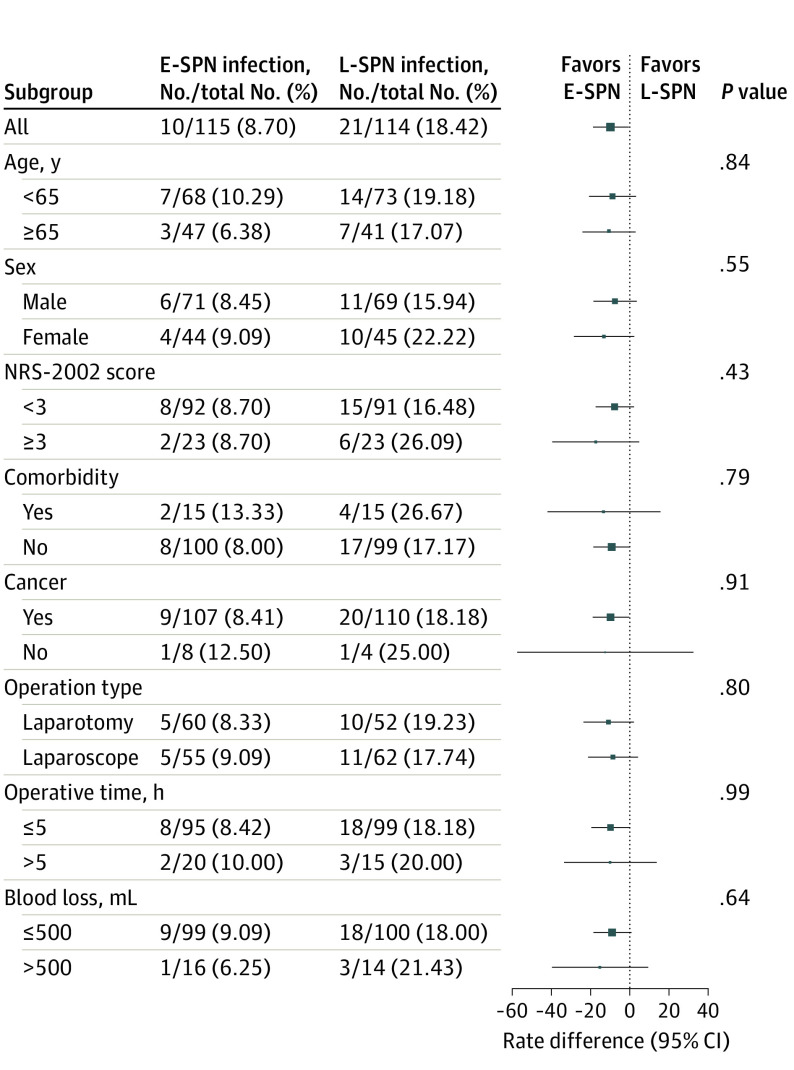
Risk Difference of Early Supplemental Parenteral Nutrition (E-SPN) vs Late Supplemental Parenteral Nutrition (L-SPN) by Prespecified Subgroups *P* value is the test of interaction between the group and each subgroup factor. Error bars indicate 95% CIs. NRS-2002 indicates Nutritional Risk Screening 2002.

Subgroup analyses of infections in the full analysis sets are shown in Figure 3. No significant differences were found in infectious complications among a priori defined subgroups. Results in all subgroups were comparable with those in the overall study population.

## Discussion

To our knowledge, this is the first multicenter randomized clinical trial to evaluate the effect of the timing for initiating SPN on the incidence of nosocomial infections in patients undergoing major abdominal surgery at high nutritional risk with poor tolerance to EN. The patients in the E-SPN group had significantly fewer nosocomial infections than those in the L-SPN group. Logically, the total energy and protein intakes were significantly higher during the intervention period (days 3-7) after surgery in the E-SPN group. In addition, E-SPN improved serum prealbumin and albumin levels before hospital discharge, which suggests some degree of improvement in nutritional risk. Therefore, E-SPN seems to be a favorable strategy to reduce nosocomial infections among patients with high nutritional risk and poor tolerance to EN after major abdominal surgery.

Postoperative gastrointestinal dysfunction often occurs in patients after abdominal surgery mainly because of gut injury, bowel wall edema, and dysmotility, which can lead to gastrointestinal intolerance and increased risk of malnutrition. The patients who experience postoperative gastrointestinal dysfunction and who cannot be nourished adequately via enteral feeding could benefit from additional nutrition via SPN to bridge the nutritional gap without symptoms of digestive intolerance. Only 3 studies (2 prospective randomized clinical trials, one in patients with esophageal cancer^33^ and one in elderly patients with gastrointestinal cancer^34^; and a retrospective cohort study^35^ in patients undergoing pancreaticoduodenectomy) have reported that early EN in combination with SPN achieved the energy target requirement and improved clinical prognosis rapidly in patients undergoing abdominal surgery compared with those receiving EN alone. However, the optimal timing of initiating SPN for patients after abdominal surgery remains unclear. Our study provides evidence that a nutritional support program of E-SPN combined with EN can reduce postoperative infection complications in patients undergoing major abdominal surgery who are at high nutritional risk and have poor tolerance to EN. Several factors might explain the between-group difference in the number of infectious complications in our trial: the trial protocol, in particular the initiation on day 3 of E-SPN, which allowed early EN to progress sufficiently so as to limit the amount of PN needed; metabolic monitoring; and appropriate selection of patients who had undergone abdominal surgery.

The previous SPN study^17^ reported that early optimization of energy provision by SPN starting 4 days after intensive care unit (ICU) admission reduced nosocomial infection in critically ill patients who failed to achieve energy goals with EN alone. In both the SPN study^17^ and our study, when the EN-alone energy supply was insufficient on day 3 (60%) or day 2 (30%), respectively, the timely prescription of SPN allowed patients to reach, without exceeding, the energy target. However, Doig et al^23^ did not find any difference the rate of infectious complications for early PN within 24 hours of ICU admission in critically ill adults with relative contraindications compared with early EN and standard nutrition. These results differ from those of the current study, maybe because of the types of diseases (high proportion of patients receiving mechanical ventilation and patients needing emergency surgery) and timing of initiating PN (day 3 after surgery in our study). However, the Early PN Trial found that early PN may significantly reduce mechanical ventilatory support time and meaningfully reduce the total cost of care,^23,36^ suggesting that early PN is clinically beneficial when EN is unsuccessful in critically ill patients.

The previous Early Parenteral Nutrition Completing Enteral Nutrition in Adult Critically Ill Patients (EPaNIC) trial compared the clinical prognosis of critically ill patients who received SPN (late-initiation PN group) initiated 8 days after entering the ICU with that of patients who had started SPN (early-initiation PN group) within 2 days.^16^ They reported that early PN increased the complications of infection significantly (26.2% vs 22.8%, *P* = .008).^16^ These results are not consistent with ours, which may be mainly attributed to the following reasons: In the EPaNIC trial, the patients received high doses of intravenous glucose during the first 2 days of the ICU stay^37^ followed by some degree of overfeeding because of the combination of EN and PN while patients were under severe metabolic stress. In our study, the supplementation of insufficient EN started on day 3 after surgery, whereas the stress and inflammatory response to surgery were already significantly decreased, a condition known to improve metabolic tolerance to exogenous energy supply.

Our study found a significant improvement in nutritional status in the E-SPN group. This finding may be attributable to the following reasons. First, E-SPN combined with EN can substantially improve energy delivery after surgery and prevent energy deficits during the initial postoperative days. Second, fewer gastrointestinal dysfunctions were found in the E-SPN group than in the L-SPN group. Previous studies^38,39^ have shown that EN combined with SPN after major abdominal surgery can effectively ensure sufficient nutrient provision and improve patients’ nutritional status, consistent with our study results.

The findings from previous studies^40,41^ indicate that E-SPN was associated with shorter ICU stay and lower mortality than L-SPN. In parallel to the lower nosocomial infection rate in the E-SPN group, fewer therapeutic antibiotic days were observed in the E-SPN group than in the L-SPN group. Results of our study further reinforce the importance of energy provision by showing that delivery of near 100% of energy supply with an E-SPN approach can effectively help decrease nosocomial infections.

### Limitations

Our study had several limitations. First, indirect calorimetry is the recommended method to measure resting energy expenditure in surgical patients according to international guidelines to individualize the energy target, whenever possible. Indirect calorimetry was unavailable in some of our centers, and we used the recommended formula.^17^ Second, this study included a select cohort of patients who had undergone major abdominal surgery and had a high nutritional risk and poor tolerance to EN, which may compromise the validity and applicability of our findings. Third, because of the nature of the study, the patients or their designated representatives and surgeons were unblinded. To reduce any potential bias, the clinical assessments were conducted by blinded nurses and investigators in charge of the data collection.

## Conclusions

In this randomized clinical trial, E-SPN was associated with reduced nosocomial infections in patients undergoing abdominal surgery. Early SPN seems to be a favorable strategy for patients at high nutritional risk and with poor tolerance to EN after major abdominal surgery to reduce the number of nosocomial infections.

## Notes

Supplement 1.
Trial Protocol

Supplement 2. **eAppendix 1.** Trial Inclusion and Exclusion Criteria **eTable 1.** Recruitment at Each Study Center **eTable 2.** Classification of Postoperative Complications **eTable 3.** Definition and Diagnostic Criteria of Infectious Complications **eTable 4.** Types and Characteristics of Surgical Procedures **eTable 5.** Mean Energy and Protein Delivery During Days 3-12 in the Intervention **eTable 6.** Distribution of Non-infectious Complications During the Intervention and Follow-up **eTable 7.** Distribution of Gastrointestinal Intolerance Complications and Parenteral Nutrition-Related Complications During the Intervention and Follow-Up **eTable 8.** Hematological Parameters at the End of the Intervention **eFigure 1.** Trial Design **eFigure 2.** Kaplan-Meier Analysis of Nosocomial Infections
eReferences

Supplement 3.
Data Sharing Statement
