# Supplementary material for: Effect of Early vs Late Supplemental Parenteral Nutrition in Patients Undergoing Abdominal Surgery: A Randomized Clinical Trial
Source: JAMA Surg. 2022 Mar 16;157(5):384–93. doi: 10.1001/jamasurg.2022.0269 (PMC8928091; doi:10.1001/jamasurg.2022.0269)
Supplement: Supplement 1. — Trial Protocol [file jamasurg-e220269-s001.pdf]

Early versus late supplemental parenteral nutrition in abdominal surgery patients: a randomized controlled clinical trial

Summary

Title: Early versus late supplemental parenteral nutrition in abdominal surgery patients: a randomized controlled clinical trial

Proposed indications: Supplemental parenteral nutrition

Study objective: In this study, we compared the effects of initiating SPN early (day 3 after surgery) and late (day 8 after surgery) on the incidence of nosocomial infections in major abdominal surgery patients at high nutritional risk and poor tolerance to enteral nutrition.

Study design: A prospective, randomized controlled, multicenter clinical trial

Study centers: 11 centers

Investigational product: Early supplemental parenteral nutrition group (Day 3 100% of energy target after surgery)

Control product: Late supplemental parenteral nutrition group (Day 8 100% of energy target after surgery)

Sample size: 230 (115 × 2)

Inclusion criteria:

1. Age: 18-80 years
2. Patients undergoing elective major abdominal surgery (elective gastric, colorectal, hepatic, and pancreatic resections for both benign and malignant disease) for nontraumatic reasons
3. Nutritional risk screening 2002 (NRS 2002) score of ≥3
4. Patients receiving EN after major abdominal surgery and unable to tolerate 30% of the energy target via enteral feeding on postoperative day 2 and are expected to have a postoperative hospital stay for longer than 7 days.

Exclusion criteria:

1. Psychiatric disorders
2. Pregnancy or breastfeeding women
3. Severe malnutrition
    a. Weight loss >10%–15% in 6 months
    b. Body mass index < 18.5

c. PG-SGA score with stage C
d. Albumin < 30 g/L

4. Unstable vital signs or unstable hemodynamics (defined as systolic blood pressure < 90 mm Hg or mean arterial pressure < 70 mm Hg after rapid infusion of 500 mL of crystal or 200 mL of gel, or 50% increase in vascular active drug infusion rate in an hour)
5. Refusal to participate in the study
6. Pre-existing infection (confirmed or strongly suspected infection episodes before randomization)
7. Mortality rates are expected to be more than 50% in 6 months with malignant or irreversibility diseases
    a. Cancer in the terminal stage
    b. HIV positive at end-stage or CD_4 ＜50/mm^3
    c. Cardiopulmonary resuscitation before cardiac arrest and nervous system function not fully recovered
    d. Class IV limitation of physical activity defined by the New York Heart Association
    e. Relying on the breathing machine because of chronic diseases
8. Life expectancy of dying patients less than 24 h
9. Refractory shock to meet any of the following conditions:
    a. Infusion rate of dopamine ＞15 μg/(kg. min)
    b. Infusion rate of dobutamine ＞15 μg/(kg .min)
    c. Infusion rate of epinephrine and norepinephrine＞30 μg/min
    d. Infusion rate of phenylephrine ＞50 μg/min
    e. Infusion rate of milrinone ＞0.5 μg/kg/min
    f. Infusion rate of vasopressin ＞0.04 U/min
    g. Inter-aortic balloon pump
10. Hepatic insufficiency (alanine/aspartate transaminase/bilirubin 200% above the normal range)
11. Renal insufficiency (creatinine 200% above the normal range)
12. Metabolic diseases (hyperthyroidism, hypothyroidism, type 1 diabetes mellitus, Wilson disease, phenylketonuria, and adrenal cortex disorders)
13. EN can reach 30% of target energy in day 2 after surgery

	14. Burn area exceeding 20% of the patient's body surface<br>15. Autoimmune diseases (systemic lupus erythematosus, Sjogren's syndrome, and dermatomyositis)<br>16. History of organ transplantation (liver, kidney, heart, and lung transplantation)<br>17. International standardization ratio more than 3.0 or platelet count＜30,000 cells/mm^3<br>18. Intracranial hemorrhage 1 month before enrolment<br>19. History of severe allergy against ingredients of enteral and parenteral nutrition<br>20. Already participated in another clinical trial<br>21. Nutritional support therapy started before enrolment<br>22. Type 2 diabetes mellitus (under intensive medical treatment or insulin treatment)
Primary endpoints:	Morbidity of infection
Secondary endpoints:	Actual calories intake and protein intake, the incidence of gastrointestinal intolerance (diarrhea, vomiting, abdominal distention, constipation, and abdominal pain) and parenteral nutrition-related complications (hyperglycemia, hypoglycemia, hyperlipidemia), postoperative non-infectious complications, length of hospital stay, hospitalization expenses, therapeutic antibiotic days (defined as days from postoperative day 3 to discharge during which a patient received at least one dose of antibiotics for actual nosocomial infection), prophylaxis antibiotic days (defined as days antibiotics were used for prophylaxis (no infection)), mechanical ventilation, mortality within 2 months after randomization, and lab tests at discharge including white blood cell, C-reactive protein, glucose, albumin, prealbumin, retinol-binding protein, transferrin, total cholesterol, triglycerides, hepatic and renal functions
Treatment duration:	5 days
Preservation of clinical data and results:	During the clinical trial, all the information of the trial should be kept by the principal investigator at each study center.

Aims

To compare the effect of initiating supplemental parenteral nutrition (SPN) early (day 3 after surgery) and late (day 8 after surgery) among abdominal surgical patients at high nutritional risk and poor tolerance to enteral nutrition.

Study Design, Materials, and Methods

Study design

PNASIT is a prospective, multicenter, randomized controlled trial with an open intervention comparing the effects of initiating early SPN with those of initiating late SPN after major abdominal surgery in whom energy targets cannot be met by enteral nutrition alone.

Setting and patient eligibility for inclusion and recruitment

The multicenter trial included 11 hospitals including Jinling Hospital, Peking Union Medical College Hospital, Chinese PLA General Hospital, Xijing Hospital, West China Hospital, Xinqiao Hospital of Chongqing, Changhai Hospital, Shanghai 10th People's Hospital, The Second Affiliated Hospital of Harbin Medical University, The Affiliated Hospital of Qingdao University, and The First Affiliated Hospital of Kunming Medical College. Upon hospital admission and 2nd day after surgery, consecutive adult in patients undergoing abdominal surgery was screened for nutrition risk by the nursing and/or physician staff, using the Nutritional Risk Screening (NRS) 2002.

Patients were eligible after surgery if they fulfilled the following inclusion criteria:

1. Age: 18-80 years
2. Undergoing elective major abdominal surgery (elective gastric, colorectal, hepatic, and pancreatic resections for both benign and malignant disease) for nontraumatic reasons
3. NRS 2002 score of ≥3
4. Patients receiving EN after major elective abdominal surgery and unable to tolerate 30% of the target targets via enteral feeding on postoperative day 2 and are expected to have a postoperative hospital stay for longer than 7 days.

The exclusion criteria were as follow:

1. Psychiatric disorders
2. Pregnancy or breastfeeding women
3. Malnutrition
    a. Weight loss >10%–15% in 6 months
    b. Body mass index (BMI) <18.5
    c. SGA score with stage C
    d. Albumin <30 g/L
4. Unstable vital signs or unstable hemodynamics (defined as systolic blood pressure <90 mm Hg or mean arterial pressure <70 mm Hg after rapid infusion of 500 mL of crystal or 200 mL of gel, or 50% increase in vascular active drug infusion rate in an hour)
5. Refusal to participate in the study
6. Pre-existing infection (confirmed or strongly suspected infection episodes before randomization)
7. Pre-existing condition with expected 6-month mortality >50%
    a. Cancer in the terminal stage
    b. HIV positive at end-stage or CD4 ＜50/mm^3
    c. Cardiopulmonary resuscitation before cardiac arrest and nervous system function not fully recovered
    d. Class IV limitation of physical activity defined by the New York Heart Association

   e. Relying on the breathing machine because of chronic diseases

8. Life expectancy less than 24 h of dying patients
9. Refractory shock to the receipt of vasopressors at the following doses:
   a. dopamine ＞15 μg/(kg. min)
   b. dobutamine ＞15 μg/(kg. min)
   c. epinephrine and norepinephrine ＞30 μg/min
   d. phenylephrine ＞50 μg/min
   e. milrinone ＞0.5 μg/(kg. min)
   f. vasopressin ＞ 0.04 U/min
   g. receipt inter-aortic balloon pump
10. Hepatic insufficiency (defined as alanine/aspartate transaminase/bilirubin 200% above the normal range)
11. Renal insufficiency (defined as creatinine 200% above the normal range)
12. Metabolic diseases (hyperthyroidism, hypothyroidism, type 1 diabetes mellitus, Wilson disease, phenylketonuria, and adrenal cortex disorders)
13. EN reaching 30% of target energy on day 2 after surgery
14. Burn area exceeding 20% of the patient's body surface
15. Autoimmune diseases (systemic lupus erythematosus, Sjogren's syndrome, and dermatomyositis)
16. History of organ transplantation (liver, kidney, heart, and lung transplantation)
17. International standardization ratio more than 3.0 or platelet count ＜30,000 cells/mm^3
18. Intracranial hemorrhage 1 month before enrolment
19. History of severe allergy against ingredients of enteral nutrition and parenteral nutrition
20. Previous enrolment in another study within the same hospital admission
21. Nutritional support therapy before enrolment
22. Diabetes mellitus (under intensive medical treatment or insulin treatment)

Data collection at study entry

All baseline assessments were obtained after obtaining informed consent from the patients or their legally authorized representative, but prior to the initiation of study procedures. Baseline assessments were made before surgery. If more than one value was available for this period, the value closest to the time of randomization was used. If no values were available from 24 h prior to randomization, then the values were measured during 6 h after randomization but prior to the initiation of intervention. The baseline assessments included the following:

1. Sociodemographic and baseline characteristics (age, sex, weight, and height for calculation of BMI, NRS 2002, disease diagnosis, surgical procedure, malignancy, and comorbidity)
2. The surgery duration, operative blood loss, operative characteristics (site and technique), intraoperative contamination, and the frequency and amount of homologous blood transfusions
3. Hematological index (albumin, pre-albumin, transferrin, retinol-binding protein, glucose, cholesterol, triglyceride, HDL, LDL, white blood cell, CRP, hepatic and renal function)
4. Energy targets
5. Medical diagnoses according to the ICD10-codes

During the study period, all patients were assessed daily by two physicians to evaluate nutritional

intake and whether nutritional targets were met. The actual energy and protein intakes each day in the intervention period were recorded.

Blood samples were systematically collected upon study inclusion and at discharge for later batch measurement of nutritional markers and other biomarkers. Postoperative prophylactic antibiotic therapy was administered to all patients as part of usual clinical practice. Also, hospital stay, occurrence of mechanical ventilation, intensive care unit (ICU) admission, mortality within 2 months after randomization, and frequency of gastrointestinal intolerance events (diarrhea, vomiting, abdominal distention, constipation, and abdominal pain) and parenteral nutrition-related complications (hyperglycemia, hypoglycemia, and hyperlipidemia) were recorded during the intervention.

Nutritional support protocol

The nutritional support protocol, including nutritional treatments and measures designed to evaluate gastrointestinal intolerance, was standardized as follows.

General principles of nutritional support in both study arms

The start of enteral feeding was encouraged as soon as possible after surgery and no later than 24 h. The caloric target for each participant was estimated based on the ideal body weight as 30 kcal/kg of the ideal body weight for men and 25 kcal/kg of the ideal body weight for women, and the protein requirements as 1.2 g/kg of ideal body weight on the day of surgery (day 0). This plan was initially based on oral nutritional supplements, and the patients were allowed to have an oral diet provided by the hospital kitchen according to patient preferences. Tube feeding was implemented if the patient could not tolerate oral feeding or the energy target could not be reached by oral feeding. The type of enteral feeding tube (nasogastric tube, nasointestinal tube, and jejunostomy) was determined by the patient’s primary medical team. Tube feeding started at the flow rate (in milliliters per hour) required to achieve the energy target. The enteral feeding was delivered continuously over the 24-h cycle without interruption. Eligible patients who couldn't achieve 30% of the energy requirement on a postoperative day 2 were randomized to two arms. The intervention group who received early SPN (E-SPN group) started achieving 100% of the caloric requirements via early EN combined with early supplemental parenteral nutrition on postoperative day 3. The patients in the control group who received late SPN (L-SPN group) started achieving 100% of the caloric requirements via early EN combined with late supplemental parenteral nutrition on postoperative day 8. After randomization, both groups received nutritional support for a minimum of 5 days, or until the transition to 80% of caloric requirements via an enteral feeding, or until hospital discharge.

Actual energy delivery was monitored regularly by comparing with the predefined daily calorie targets. Any interruption in energy delivery was reported to the physicians in charge. Usually, nutritional support was not interrupted while transporting the patient. However, when EN was interrupted (for example, a specific gastrointestinal or radiological investigation for a gastrointestinal intolerance event), the flow rate was not increased to compensate for the interruption. All patients were kept in the semi-recumbent supine position.

Nutritional formulas

All patients received commercially available standardized enteral nutrition formulas. The EN products comprising polymeric formulas were routinely prescribed at all hospitals, which contained 1 kcal/mL of energy (16% proteins, 35% lipids, and 49% carbohydrates). PN formulas consisted of 0.88 kcal/mL of energy (15% proteins, 40% lipids [20% long-chain triglycerides], and 45% carbohydrates) and

supplemental vitamins and minerals. PN infusion was administered via the peripheral or central veins.

Additional intakes

Patients who were assigned to the L-SPN group received 5% glucose saline and saline solution in a volume equal to that of the SPN administered in the early initiation group in order to provide adequate hydration, with the delivered volume of EN taken into account according to the needs of each participant as assessed by the physicians in charge and in compliance with standard practice at each center.

Complications possibly related to nutrition management

In order to monitor the quality of the enteral and parenteral nutrition management during the study we will register all known complications possibly related to them.

Complications possibly related to enteral feeding

Gastrointestinal intolerance: diarrhea, vomiting, abdominal distention, constipation, and abdominal pain.

Complicated insertion of feeding tubes: nasal bleeding.

Mechanical complications: feeding tube displacement or obstruction.

Complications possibly related to parenteral feeding

Mechanical complications: occlusion and dislodging of central venous catheters.

Clinical complications: pneumothorax, hemothorax, and subclavian artery, liver function abnormalities, hyperlipidemia, hyperglycemia, central line replacement due to suspicion of blood stream-related infections (BSRI).

Parenteral nutrition-related complications

Including hyperglycemia, hypoglycemia, and hyperlipidemia. Hypoglycemia resistance to parenteral glucose administration as a result of prolonged hypocaloric feeding will be considered a serious adverse event.

The principles implemented for blood glucose monitoring and management are as follows:

- Regular blood glucose levels were monitored once a day before surgery and three times a day after surgery.
- In the early stage of supplemental parenteral nutrition, blood glucose levels were monitored every 6 h and twice a day after stable blood glucose levels were achieved.
- The diagnostic criterion for parenteral nutrition-related hyperglycemia was the measurement of blood glucose levels >180 mg/dL (10 mmol/L).
- The number of occurrences of hyperglycemia and hypoglycemia during supplemental parenteral nutrition intervention was recorded.

Gastrointestinal intolerance monitoring

Gastrointestinal tolerance was assessed based on episodes of significant vomiting (defined as the expulsion of gastric contents from the oro- or nasopharynx), abdominal distention (defined as the presence of a tense or rigid abdomen with guarding or rebound on examination), diarrhea (more than 300 mL of liquid stool or more than four loose stools per day), constipation (no stool for more than 6 days), and abdominal pain (defined as feeling pain anywhere between the chest and the groin). Patients were monitored for gastrointestinal intolerances by the medical team every 6 h when the rates were stable and every 2 h for any gastrointestinal intolerance until it disappeared. They could evaluate patients more frequently if warranted by the clinical condition. A predefined protocol was used to

manage gastrointestinal intolerance. In cases of vomiting, a prokinetic agent was administered first after confirming the absence of any contraindications. The decision to use prokinetic agents was left to the discretion of the medical team. The prokinetic agent was continued until EN at the highest prescribed flow rate was well tolerated for 24 h. Then, the prokinetic agent was discontinued. If the intolerance persisted despite prokinetic therapy, feeds were held for 2 h. Feeding was restarted at a rate 10 mL/h slower than previously after 2 h and confirmation of the resolution of the signs of intolerance. When abdominal distention occurred, the enteral feeding rate was decreased by 10 mL/h. Further, the patient was reevaluated every 2 h until distention improved, and then the rates were increased to previous rates. For diarrhea, the medical team was encouraged to minimize offending medications and initiate pharmacologic treatments. If diarrhea became worse, then enteral feeding was interrupted. When abdominal pain was reported, EN was continued and reevaluated every 2 h until it improved. If the abdominal pain became worse, EN was stopped and an abdominal radiograph examination was performed. After confirmation of the resolution of the signs of intolerance, feeds were restarted at the highest prescribed flow rate. The primary team interrupted or halted enteral feeding if it was thought necessary for the patient's health.

Study endpoints

All patients were daily assessed until hospital discharge and contacted after discharge via telephone for a structured interview by blinded study nurses to assess mortality within 2 months after randomization.

Primary outcomes

The primary outcome was morbidity of infection that occurred between postoperative day 3 and discharge. Infections were defined according to the Centers for Disease Control and Prevention. Six infection categories were defined: bloodstream infections (laboratory-confirmed bloodstream infections and clinical sepsis), pneumonia (clinically defined pneumonia, pneumonia with specific laboratory findings, ventilator-associated pneumonia, and other lower respiratory tract infections), urinary tract infections (symptomatic urinary tract infection and other infections of the urinary tract), surgical site infections (superficial or deep incisional surgical site infections), abdominal infections (organ/space surgical site infections at the intraabdominal specific site), and other infections (skin and soft tissue infection)

Secondary outcomes

The secondary endpoints were the actual caloric and protein intakes each day during an intervention, length of hospital stay, hospitalization expenses, the occurrence of mechanical ventilation, ICU admission, postoperative noninfectious complications, therapeutic antibiotic days (defined as days from postoperative day 3 to discharge during which a patient received at least one dose of antibiotics for actual nosocomial infection), prophylaxis antibiotic days (defined as days antibiotics were used for prophylaxis (no infection)), mortality within 2 months after randomization, and concentrations of albumin, pre-albumin, transferrin, and retinol-conjugated protein, white blood cell, C-reactive protein, glucose, cholesterol, triglyceride, LDL, HDL, AST, ALT, ALP, TBiL, urea nitrogen, and creatinine at discharge. Gastrointestinal intolerance events and their frequency during the intervention were as follows: diarrhea (more than 300 mL of liquid stool or more than four loose stools per day), vomiting (defined as the expulsion of gastric contents from the oro-or nasopharynx), abdominal distention (defined as the presence of a tense or rigid abdomen with guarding or rebound on examination), constipation (no stool for more than 6 days), and abdominal pain (defined as pain anywhere between

the chest and the groin) and parenteral nutrition-related complications (including hyperglycemia, hypoglycemia, hyperlipidemia and so on) during the intervention.

Randomization

Eligible patients were randomized in a 1:1 fashion into the intervention group or the control group according to a computer-generated, randomization scheme using SAS 9.4 Statistical Analysis System (SAS Institute Inc). The randomization was performed using the random block design with block sizes 2, 4, 6, and 8, and stratified for participating centers. A sufficient number of sealed, sequentially numbered, opaque envelopes were prepared for each center. A randomized competitive enrolment among different centers was implemented. The participants at each center regularly reported, and no new participants were enrolled once the targeted sample size was reached. Staff responsible for randomization will only be responsible for the assignment of random groups and will not be involved in any specific trial operations.

Blinding

Blinding the clinical staff to the patient's assigned early or late SPN protocol was not feasible. All participants and investigators were aware of the group assignment, but the follow-up assessments were performed by trained physicians and nurses who were blinded to the patient's assignment. Furthermore, the statisticians are blinded to the treatment groups during the data analysis.

Informed consent

The research physician investigator explained the objectives of this study and its potential risks and benefits to the patient or his/her surrogate decision-maker. Once they agreed, written consent was obtained. The participants could withdraw from the study at any time without any impact on treatment.

Ethical considerations

This clinical study was conducted in accordance with the Declaration of Helsinki and all applicable Chinese laws and regulations on clinical trial conduct. The study was approved by the institutional ethics committees (IEC) of the participating hospitals and conducted in compliance with the protocol approved by the IEC and according to the international conference of harmonization (ICH)-Good Clinical Practice standards. Before initiating the study, each of the investigators had written and dated approval/favorable opinion from their concerned IEC for the study protocol (and any amendments), written informed consent form, consent form updates, patient recruitment procedures (e.g., advertisements), and any other written information to be provided to patients.

All enrolled participants were asked to provide written informed consent. For patients who could not do so due to hypothermia or their acute medical condition, patients' next to kin signed an assent form to state the presumptive will of the patient. The patient was included in the study only after these informed consent procedures.

Statistical analysis

Sample size calculation

The incidence of postoperative infectious complications reached 10%-30% among patients undergoing abdominal surgery. The infection rate was assumed to be 25% in the group that received L-SPN and 10% in the E-SPN group. The total sample size of 220 was calculated using power analysis & sample size 15.0 software. With a two-tailed type, I error rate of 5%. The sample size was increased to 230 to allow for withdrawal and loss to follow-up.

Missing data

Since the primary outcome was the rate of infection, all randomized participants who died during hospitalization or withdrew from the study were assigned to occurring infection.

Analysis populations

Intent-to-treat (ITT) set: This set included participants randomized to receive E-SPN in the intervention arm and those randomized to receive L-SPN in the control arm.

Full analysis set (FAS): This set was the same as the ITT population except participants who did not receive any intervention. This population was the primary population for the primary outcome.

Per-protocol set (PPS): This was a subset of the ITT population, including participants who did not violate the inclusion/exclusion criteria or experience significant protocol deviations. PP analyses were conducted for all secondary outcomes. Primary outcome analyses were also repeated in the PP population as sensitivity analyses.

Safety set (SS): The set of participants to be summarized was usually defined as randomized participants who received at least one dose of investigation and a one-time safety assessment after treatment. The incidence of gastrointestinal intolerance events and other adverse events (the principal safety outcome) was compared among participants in the safety set.

Throughout the analyses, statistical significance was accepted at a ***p-value*** of .05. All analyses were performed using SAS software version 9.4 (SAS Institute Inc).

Demographics and baseline characteristics

Specified demographics and baseline characteristics were presented for the E-SPN and L-SPN groups. Continuous variables, including age, weight, body mass index, hematological index (albumin, pre-albumin, transferrin, retinol-binding protein, white blood cell, C-reactive protein, glucose, cholesterol, triglyceride, LDL, HDL, AST, ALT, ALP, TBiL, urea nitrogen, and creatinine) and energy goal, were summarized with their means and standard deviation (normal distribution) or medians and interquartile range (non-normal distribution). Categorical data (sex, NRS 2002, operative site, surgical procedure, malignancy, surgical stages, intraoperative contamination, duration of operative time, blood loss, blood transfusion, and comorbidity) were described by the number and percentage of patients.

Demographic and baseline variables were compared between the two groups with appropriate statistical tests to assess the balance of randomization. The Shapiro-Wilk test was used to test the normality of continuous variables. The student t-test was used for continuous variables, and the Mann–Whitney U test was used to compare ordinal data. The categorical data were performed using the Chi-square test or Fisher’s exact test.

Analyses of efficacy outcome

Primary outcome analyses

The main analysis was based on the ITT principle. The primary outcome was the occurrence of all-cause infection between postoperative day 3 and discharge. Infections were defined according to the Centers for Disease Control and Prevention. Six infection categories were defined: bloodstream infections (laboratory-confirmed bloodstream infections and clinical sepsis), pneumonia (clinically defined pneumonia, pneumonia with specific laboratory findings, ventilator-associated pneumonia, and other lower respiratory tract infections), urinary tract infections (symptomatic urinary tract infection and other infections of the urinary tract), surgical site infections (superficial or deep incisional surgical site infections), abdominal infections (organ/space surgical site infections at the intraabdominal

specific site), and other infections (skin and soft tissue infection). Meanwhile, the difference and its two-sided 95% confidence interval (CI) between the two groups were calculated. Primary outcome analyses were also performed in FAS and PPS populations. In addition, the rate of nosocomial infections in a time-to-event analysis was reported with the use of Kaplan–Meier plots, and the difference between the two groups was tested by log-rank test. Cox proportional hazards model was used to estimate the hazards ratio (HR) and corresponding 95% confidence intervals (CIs). The subgroup analyses for the primary outcome included the following variables: age (<65 vs. ⩾65), sex (male vs. female), NRS2002 score (3 vs. ⩾4), comorbidity (yes vs. no), cancer (yes vs. no), operation type (laparotomy vs. laparoscope), operation time (⩽5h vs. >5h) and blood loss (⩽500ml vs. >500ml).

Secondary efficacy analyses

Secondary outcome measures included the following:

1. Daily energy received from enteral nutrition (kcal per day)
2. Daily energy received from parenteral nutrition (kcal per day)
3. Daily energy received from total nutrition support (kcal per day)
4. Daily energy received from total nutrition support (kcal/kg per day)
5. Daily protein from enteral nutrition (g per day)
6. Daily protein from parenteral nutrition (g per day)
7. Daily protein from total nutrition support (g per day)
8. Daily protein from total nutrition support (g/kg per day)
9. Length of hospital stays
10. Occurrence of mechanical ventilation
11. ICU admission
12. Hospitalization costs
13. Total antibiotic days (prophylactic antibiotic days and therapeutic antibiotic days)
14. Adverse effects of artificial nutrition (GI intolerance complications and parenteral nutrition-related complications)
15. All-cause mortality within 2 months after randomization
16. Nutritional indicators (albumin, pre-albumin, transferrin, and retinol-binding protein) at discharge
17. Hepatic and renal function(ALT, AST, ALP, TBiL, blood urea nitrogen, and serum creatinine) at discharge
18. Metabolism-related index (blood glucose, total cholesterol, triglyceride, HDL, and LDL) at discharge
19. Inflammatory biomarkers (white blood cell and C-reactive protein) at discharge
20. Postoperative non-infectious complication (anastomotic leak, wound dehiscence, bleeding, intestinal obstruction, hemoperitoneum, arrhythmia, hepatic dysfunction, renal dysfunction, respiratory failure, pleural effusion, and atelectasis. etc.)

Comparisons of secondary outcomes between two arms were performed using the Chi-square test or Fisher's exact test as appropriate (#10, #11, #14, #15, and #20). The Student's test or Mann–Whitney *U* test was used in the analyses of continuous and ordinal outcome data (#1, #2, #3, #4, #5, #6, #7, #8, #9, #12, #13, #16, #17, #18, and #19) between the two groups.

Safety analyses

The safety outcome measure was the gastrointestinal intolerance events and parenteral nutrition-related complications including the following:

1. Vomiting (expulsion of gastric contents from the oro- or nasopharynx)
2. Abdominal distention (presence of a tense or rigid abdomen with guarding or rebound on examination)
3. Constipation (no stool for more than 6 days)
4. Diarrhea (more than 300 mL of liquid stool or more than four loose stools per day)
5. Abdominal pain (feeling pain anywhere between the chest and the groin)
6. Hyperglycemia (target level for serum glucose of < 180 mg/dL (10 mmol/L))
7. Hypoglycemia (target level for serum glucose of < 90 mg/dL (3.9 mmol/L))
8. Hyperlipidemia (triglyceride level should not exceed 400 mg/dL (4.5 mmol/L) during infusion)

The number and proportion of the gastrointestinal intolerance events and other adverse events in the two groups were reported. Since the safety outcome measures were all categorical data, the Chi-square test or Fisher's exact test was used to analyze the differences between the two treatment groups as appropriate.

Administrative and legal aspects

Electronic data collection will be used. Data will be collected in an anonymous, CRF, unambiguously linked to the source file. The sponsor will provide direct access to the CRF, the source data and the study master file for monitoring, Independent Ethics committee review and regulatory inspection. The investigator will establish an independent data and safety monitoring board to oversee the study conduct and review blinded safety data. The investigator appointed two monitors. The monitor will verify that the trial is performed in accordance with the protocol as described in the China National Medical Products Administration as well as the Declaration of Helsinki. Monitoring will be performed and will be reported following the sponsor's SOPs.

Study personnel

A list of all study personnel and investigators will be updated in the study master file.

All investigators not directly involved in the patients' care will be blinded to treatment allocation:

Statisticians

Microbiologists

Pathologists

….

Appendix 1: Trial design

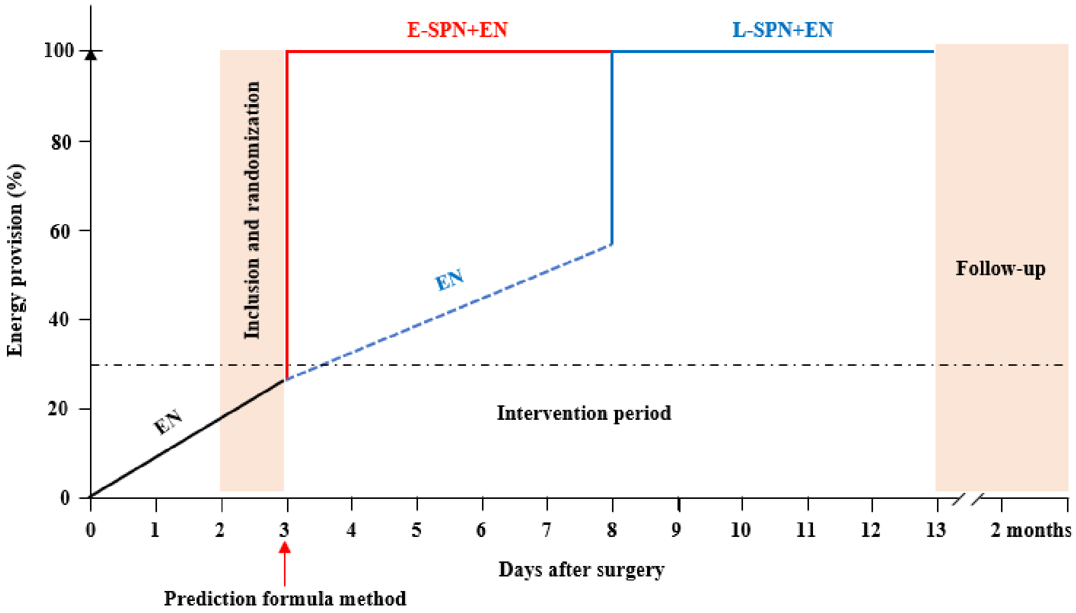

The black solid line shows the potential progression of EN in all patients before inclusion into the trial (Day 2), and the red line shows the energy delivery in patients on EN with E-SPN during the intervention period (Days 3-12), resulting in the potential prescription of 100% of the energy target (determined by prediction formula, 2 days after surgery). The blue broken line shows the potential energy provision for patients remaining on EN only (Days 3-7), and the blue solid line the energy delivery in patients on EN with L-SPN during the intervention period (Days 8-12), resulting in the potential prescription of 100% of the energy target. EN=enteral nutrition, E-SPN=early supplemental parenteral nutrition, L-SPN=late supplemental parenteral nutrition.

Appendix 2: Protocol for nutritional screening

**Nutritional risk screening(NRS 2002)**			
**Impaired nutritional status**		**Severity of disease (=increase in requirements)**	
Absent **Score 0**	Normal nutritional status	Absent **Score 0**	Normal nutritional requirements
Mid **Score 1**	Wt loss > 5% in 3 mths or Food intake below 50-75% of normal requirement in preceding week	Mid **Score 1**	Hip fracture* Chronic patients, in particular with acute complications: cirrhosis*, COPD*, *Chronic hemodialysis, diabetes, oncology*
Moderate **Score 2**	Wt loss > 5% in 2 mths or BMI 18.5 - 20.5 + impaired general condition or Food intake 25-50% of normal requirement in preceding week	Moderate **Score 2**	Major abdominal surgery* Stroke* *Severe pneumonia, hematological malignancy*
Severe **Score 3**	Wt loss > 5% in 1 mth (> 15% in 3 mths) or BMI < 18.5 + impaired general condition or Food intake 0-25% of normal requirement in preceding week in preceding week	Severe **Score 3**	Head injury* Bone marrow transplantation* *Intensive care patients (APACHE > 10).*
**Score:**	**+**	**Score:**	**= Total score**
Age	If 70 years: add 1 to total score above	= age adjusted total score	
**Score 3:** the patient is nutrionally at-risk and a nutritional care plan is initiated.<br>**Score < 3:** weekly re-screening of the patient. E.g., if the patient is scheduled for a major operation, a preventive nutritional care plan is considered to avoid the associated risk status.			

NRS-2002 is based on an interpretation of available randomized clinical trials.

*Indicates that a trial directly supports the categorization of patients with that diagnosis. Diagnosis shown in italics is based on the prototypes given below.

Nutritional risk is defined by the present nutritional status and risk of impairment of present status, due to increased requirements caused by stress metabolism of the clinical condition.

A nutritional care plan is indicated in all patients who are (1) severely undernourished (score=3), or (2) severely ill (score=3), or (3) moderately undernourished + mildly ill (score 2+1), or (4) mildly undernourished + moderately ill (score 1+2).

Prototypes for severity of disease

Score=1: a patient with chronic disease, admitted to hospital due to complications. The patient is weak but out of bed regularly. Protein requirement is increased but can be covered by oral diet or supplement in most cases.

Score=2: a patient confined to bed due to illness, e.g., following major abdominal surgery. Protein

requirement is substantially increased, but can be covered, although artificial feeding is required in many cases.

Score=3: a patient in intensive care with assisted ventilation etc. Protein requirement is increased and cannot be covered even by artificial feeding. Protein breakdown and nitrogen loss can be significantly attenuated.

Appendix 3: Formula for calculating Ideal Body Weight (IBW)

Female：IBW=45.5+[0.91*(Height cm -152.4)]

Male：IBW=50+[0.91*(Height cm -152.4)]

Corrected Ideal body weight

If　27≥BMI≥18.5, IBW

If　BMI > 27, IBW *1.2
