# Supplementary material for: Effect of Early vs Late Supplemental Parenteral Nutrition in Patients Undergoing Abdominal Surgery: A Randomized Clinical Trial
Source: JAMA Surg. 2022 Mar 16;157(5):384–93. doi: 10.1001/jamasurg.2022.0269 (PMC8928091; doi:10.1001/jamasurg.2022.0269)
Supplement: Supplement 2. — eAppendix 1. Trial Inclusion and Exclusion Criteria eTable 1. Recruitment at Each Study Center eTable 2. Classification of Postoperative Complications eTable 3. Definition and Diagnostic Criteria of Infectious Complications eTable 4. Types and Characteristics of Surgical Procedures eTable 5. Mean Energy and Protein Delivery During Days 3-12 in the Intervention eTable 6. Distribution of Non-infectious Complications During the Intervention and Follow-up eTable 7. Distribution of Gastrointestinal Intolerance Complications and Parenteral Nutrition-Related Complications During the Intervention and Follow-Up eTable 8. Hematological Parameters at the End of the Intervention eFigure 1. Trial Design eFigure 2. Kaplan-Meier Analysis of Nosocomial Infections eReferences [file jamasurg-e220269-s002.pdf]

Supplementary Online Content

Gao X, Liu Y, Zhang L, et al. Effects of early vs late supplemental parenteral nutrition in patients undergoing abdominal surgery: a randomized clinical trial. *JAMA Surg*. Published online March 16, 2022. doi:10.1001/jamasurg.2022.0269

eAppendix. Trial Inclusion and Exclusion Criteria
eTable 1. Recruitment at Each Study Center
eTable 2. Classification of Postoperative Complications
eTable 3. Definition and Diagnostic Criteria of Infectious Complications
eTable 4. Types and Characteristics of Surgical Procedures
eTable 5. Mean Energy and Protein Delivery During Days 3-12 in the Intervention
eTable 6. Distribution of Non-infectious Complications During the Intervention and Follow-up
eTable 7. Distribution of Gastrointestinal Intolerance Complications and Parenteral Nutrition-Related Complications During the Intervention and Follow-Up
eTable 8. Hematological Parameters at the End of the Intervention
eFigure 1. Trial Design
eFigure 2. Kaplan-Meier Analysis of Nosocomial Infections
eReferences

This supplementary material has been provided by the authors to give readers additional information about their work.

© 2022 American Medical Association. All rights reserved.

eAppendix. Trial inclusion and exclusion criteria

Inclusion criteria

1. Age: 18-80 years
2. Undergoing elective major abdominal surgery (including elective gastric, colorectal, hepatic, and pancreatic resections for both benign and malignant disease) for nontraumatic reasons
3. NRS 2002 score of ≥3
4. Patients receiving EN after elective major abdominal surgery and unable to tolerate 30% of the energy target via enteral feeding on postoperative day 2 and are expected to have a postoperative hospital stay for longer than 7 days.

Exclusion criteria

Patients were not eligible for enrolment if they met one or more of the following criteria:

1. Psychiatric disorders
2. Pregnancy or breast-feeding women
3. Preoperative severe malnutrition (defined as non-volitional weight loss >10%-15% in 6 months, or BMI<18.5kg/m^2, or PG-SGA score with stage C, or albumin < 30g/L)
4. Unstable vital signs or unstable hemodynamics (defined as systolic blood pressure < 90 mmHg or mean arterial pressure < 70 mmHg after rapid 500 ml crystal or 200 ml gel infusion, or the 50% increase of vascular active drug infusion rate in an hour)
5. Refuse to participate in this study
6. Pre-existing infection (confirmed or strongly suspected infections before randomization)
7. Pre-existing condition with expected six months mortality >50% (i.e., cancer in the terminal stage, HIV positive at end-stage or CD4< 50/mm^3, cardiopulmonary resuscitation (CPR) before cardiac arrest and nervous system function not fully recovery, Class IV limitation of physical activity defined by New York heart association, rely on breathing machine because of chronic diseases)
8. Dying patients whose life expectancy is less than 7 days
9. Refractory shock to receipt of vasopressors at the following doses: dopamine >15 ug/kg/min, dobutamine >15ug/kg/min, epinephrine and norepinephrine >30ug/min, phenylephrine >50ug/min, milrinone >0.5ug/kg/min, vasopressin >0.04 U/min or receipt inter aortic balloon pump (IABP)
10. Hepatic insufficiency (defined as alanine or aspartate transaminase/bilirubin 200% above normal range)
11. Renal insufficiency (defined as creatinine 200% above normal range)
12. Metabolic diseases (hyperthyroidism, hypothyroidism, type 1 diabetes mellitus, Wilson disease, phenylketonuria, and adrenal cortex disorders)
13. EN can reach 30% of target energy on day 2 after surgery
14. Burn area exceeding 20% of the patient's body surface
15. Autoimmune diseases (systemic lupus erythematosus, Sjogren's syndrome, and dermatomyositis)
16. History of organ transplantation (liver, kidney, heart, and lung transplantation)
17. International standardization ratio (INR) more than 3.0 or platelet count<30000 cells/mm^3
18. Intracranial hemorrhage one month before enrolment
19. History of severe allergy against ingredients of enteral and parenteral nutrition
20. Previous enrollment in other studies within the same hospital admission
21. Nutritional support therapy started before enrolment
22. Type 2 diabetes mellitus (under intensive medical treatment or insulin treatment)

© 2022 American Medical Association. All rights reserved.

eTable 1. Recruitment at each study center

Participating Centres	Patients included	
	N	(%)
Jinling Hospital	6	2.61
Peking Union Medical College Hospital	11	4.78
Chinese PLA General Hospital	3	1.30
Xijing Hospital	17	7.39
West China Hospital	1	0.43
Xinqiao Hospital of Chongqing	24	10.43
Changhai Hospital	57	24.78
Shanghai 10th People's Hospital	11	4.78
The Second Affiliated Hospital of Harbin Medical University	28	12.17
The Affiliated Hospital of Qingdao University	30	13.04
The First Affiliated Hospital of Kunming Medical College	42	18.26

© 2022 American Medical Association. All rights reserved.

eTable 2. Classification of postoperative complications

Classification complications
**Major infectious**
Pneumonia
Abdominal infection
Bloodstream infection
Septic shock
**Minor infectious**
Surgical site infection
Urinary tract infection
Other infections (skin and soft tissue infections)
**Major noninfectious**
Anastomotic leak
Wound dehiscence
Gastrointestinal complications
Bleeding
Perforation, obstruction, and ischemia
Pancreatitis
Cardiovascular complications
Myocardial infarction
Cardiopulmonary arrest
Stroke
Arrythmia
Pulmonary embolus
Hemoperitoneum
Respiratory failure
Renal failure
Renal dysfunction
Hepatic dysfunction
**Minor noninfectious**
Pleural effusion
Atelectasis

© 2022 American Medical Association. All rights reserved.

eTable 3. Definition and diagnostic criteria of infectious complications

Complication	Definition and diagnostic criteria
**Bloodstream infection**	**LCBI-Laboratory-confirmed bloodstream infection[1]**<br>LCBI criteria 1 and 2 may be used for patients of any age<br>LCBI must meet at least 1 of the following criteria:<br>1. Patient has a recognized pathogen cultured from 1 or more blood cultures and organism cultured from blood is not related to an infection at another site.<br>2. Patient has at least 1 of the following signs or symptoms: fever (>38°C), chills, or hypotension and signs and symptoms and positive laboratory results are not related to an infection at another site and common skin contaminant (ie, diphtheroids [Corynebacterium spp], Bacillus [not B anthracis] spp, Propionibacterium spp, coagulase-negative staphylococci [including S epidermidis], viridans group streptococci, Aerococcus spp, Micrococcus spp) is cultured from 2 or more blood cultures drawn on separate occasions.
	**Sepsis was defined as[2, 3]:**<br>Defined focus of infection (Defined focus of infection was indicated by either an organism grown in blood or sterile site, or an abscess or infected tissue (e.g., pneumonia, peritonitis, urinary tract, vascular line infection, soft tissue, etc.)).<br>AND at least two systemic inflammatory response syndrome (SIRS) criteria:<br>1. Core temperature >38°C or <36°C. (Core temperature was rectal or tympanic). If oral, inguinal or axillary temperatures were used, 0.5°C were added to the measured value.<br>2. Heart rate >90/min. If patient had an atrial arrhythmia, record the ventricular rate. If patients have a known medical condition or are receiving treatment that would prevent tachycardia (for example, heart block or beta blockers), they must meet two of the remaining three SIRS criteria.<br>3. Respiratory rate >20 breaths per min or a PaCO2 <32 mmHg (4.3 kPa) or mechanical ventilation for an acute process.<br>4. White Blood Cell (WBC) count of >12 x 10^9/L or <4 x 10^9/L.
**Pneumonia**	**PNU1-clinically defined pneumonia[1]**<br>**Radiology**<br>Two or more serial chest radiographs with at least 1 of the following:<br>1. New or progressive and persistent infiltrate<br>2. Consolidation

© 2022 American Medical Association. All rights reserved.

	3. Cavitation<br>**Signs/Symptoms**<br>For any patient, at least 1 of the following:<br>1. Fever (>38℃) with no other recognized cause<br>2. Leukopenia (<4000 WBC/mm3) or leukocytosis (≥12,000 WBC/mm3)<br>3. For adults≥70 years, altered mental status with no other recognized cause<br>And at least 2 of the following:<br>1. New onset of purulent sputum or change in character of sputum or increased respiratory secretions or increased suctioning requirements<br>2. New onset or worsening cough, or dyspnea, or tachypnea<br>3. Rales or bronchial breath sounds<br>4. Worsening gas exchange (eg, O2 desaturations [eg, PaO2/FiO2≤240], increased oxygen requirements, or increased ventilator demand<br>**PNU2-Pneumonia with specific laboratory findings was defined as[1] VAP-Ventilator-associated pneumonia[4] and other lower respiratory tract infections were defined as[1]**
**Urinary tract infection**	**SUTI-Symptomatic urinary tract infection[1]**<br>A symptomatic urinary tract infection must meet at least 1 of the following criteria:<br>1. Patient has at least 1 of the following signs or symptoms with no other recognized cause: fever (>38℃), urgency, frequency, dysuria, or suprapubic tenderness and patient has a positive urine culture, that is,≥10^5microorganisms per cc of urine with no more than 2 species of microorganisms.<br>2. Patient has at least 2 of the following signs or symptoms with no other recognized cause: fever (>38℃), urgency, frequency, dysuria, or suprapubic tenderness<br>And at least 1 of the following:<br>1. positive dipstick for leukocyte esterase and/ or nitrate<br>2. pyuria (urine specimen with≥10 white blood cell [WBC]/mm^3 or≥3 WBC/high power field of unspun urine)<br>3. organisms seen on Gram's stain of unspun urine<br>4. at least 2 urine cultures with repeated isolation of the same uropathogen (gram negative bacteria or Staphylococcus saprophyticus) with≥10^2 colonies/mL in nonvoided specimens.<br>5. ≤10^5 colonies/mL of a single uropathogen (gram-negative bacteria or S saprophyticus) in a patient being treated with an effective antimicrobial agent for a urinary tract infection

© 2022 American Medical Association. All rights reserved.

	6. physician diagnosis of a urinary tract infection<br>7. physician institutes appropriate therapy for a urinary tract infection.<br>**OUTI-Other infections of the urinary tract (kidney, ureter, bladder, urethra, or tissue surrounding the retroperitoneal or perinephric space)[1]**<br>Other infections of the urinary tract must meet at least 1 of the following criteria:<br>1. Patient has organisms isolated from culture of fluid (other than urine) or tissue from affected site.<br>2. Patient has an abscess or other evidence of infection seen on direct examination, during a surgical operation, or during a histopathologic examination.<br>3. Patient has at least 2 of the following signs or symptoms with no other recognized cause: fever (>38℃), localized pain, or localized tenderness at the involved site<br>And at least 1 of the following:<br>1. purulent drainage from affected site<br>2. organisms cultured from blood that are compatible with suspected site of infection<br>3. radiographic evidence of infection (eg, abnormal ultrasound, computerized tomography [CT] scan, magnetic resonance imaging [MRI], or radiolabel scan [gallium, technetium], etc)<br>4. physician diagnosis of infection of the kidney, ureter, bladder, urethra, or tissues surrounding the retroperitoneal or perinephric space<br>5. physician institutes appropriate therapy for an infection of the kidney, ureter, bladder, urethra, or tissues surrounding the retroperitoneal or perinephric space.
**Surgical site infection**	**SIS-Superficial incisional surgical site infection[1]**<br>A superficial incisional SSI (SIS) must meet the following criterion:<br>Infection occurs within 30 days after the operative procedure and involves only skin and subcutaneous tissue of the incision<br>And patient has at least 1 of the following:<br>1. purulent drainage from the superficial incision<br>2. organisms isolated from an aseptically obtained culture of fluid or tissue from the superficial incision<br>3. at least 1 of the following signs or symptoms of infection: pain or tenderness, localized swelling, redness, or heat, and superficial incision is deliberately opened by surgeon and is culture positive or not cultured. A culture-negative finding does not meet this criterion.

© 2022 American Medical Association. All rights reserved.

	4. diagnosis of superficial incisional SSI by the surgeon or attending physician.<br>**DIS-Deep incisional surgical site infection[1]**<br>A deep incisional SSI (DIS) must meet the following criterion:<br>Infection occurs within 30 days after the operative procedure if no implant1 is left in place or within 1 year if implant is in place and the infection appears<br>to be related to the operative procedure and involves deep soft tissues (eg, fascial and muscle layers) of the incision and patient has at least 1 of the following:<br>1. purulent drainage from the deep incision but not from the organ/space component of the surgical site<br>2. a deep incision spontaneously dehisces or is deliberately opened by a surgeon and is culture-positive or not cultured when the patient has at least 1 of the following signs or symptoms: fever (>38℃), or localized pain or tenderness. A culture-negative finding does not meet this criterion.<br>3. an abscess or other evidence of infection involving the deep incision is found on direct examination, during reoperation, or by histopathologic or radiologic examination<br>4. diagnosis of a deep incisional SSI by a surgeon or attending physician.
**Abdominal infection**	**Organ/space surgical site infection at the intraabdominal specific site[1]**<br>Intraabdominal infections must meet at least 1 of the following criteria:<br>1. Patient has organisms cultured from purulent material from intraabdominal space obtained during a surgical operation or needle aspiration.<br>2. Patient has abscess or other evidence of intraabdominal infection seen during a surgical operation or histopathologic examination.<br>3. Patient has at least 2 of the following signs or symptoms with no other recognized cause: fever (>38℃), nausea, vomiting, abdominal pain, or jaundice<br>And at least 1 of the following:<br>1. organisms cultured from drainage from surgically placed drain (eg, closed suction drainage system, open drain, T-tube drain)<br>2. organisms seen on Gram's stain of drainage or tissue obtained during surgical operation or needle aspiration<br>3. organisms cultured from blood and radiographic evidence of infection (eg, abnormal findings on ultrasound, CT scan, MRI,

© 2022 American Medical Association. All rights reserved.

	or radiolabel scans [gallium, technetium, etc] or on abdominal x-ray).
**Skin and soft tissue infection**	**Skin infections must meet at least 1 of the following criteria[1]:**<br>1. Patient has purulent drainage, pustules, vesicles, or boils.<br>2. Patient has at least 2 of the following signs or symptoms with no other recognized cause: pain or tenderness, localized swelling, redness, or heat<br>And at least 1 of the following:<br>1. organisms cultured from aspirate or drainage from affected site; if organisms are normal skin flora (ie, diphtheroids [Corynebacterium spp], Bacillus [not Banthracis] spp, Propionibacterium spp, coagulase-negative staphylococc [including S epidermidis], viridans group streptococci, Aerococcus spp, Micrococcus spp), they must be a pure culture<br>2. organisms cultured from blood<br>3. positive antigen test performed on infected tissue or blood (eg, herpes simplex, varicella zoster, H influenzae, N meningitidis)<br>4. multinucleated giant cells seen on microscopic examination of affected tissue<br>5. diagnostic single antibody titer (IgM) or 4-fold increase in paired sera (IgG) for pathogen<br>**Soft tissue (necrotizing fascitis, infectious gangrene, necrotizing cellulitis, infectious myositis, lymphadenitis, or lymphangitis)[1]**<br>Soft tissue infections must meet at least 1 of the following criteria:<br>1. Patient has organisms cultured from tissue or drainage from affected site.<br>2. Patient has purulent drainage at affected site.<br>3. Patient has an abscess or other evidence of infection seen during a surgical operation or histopathologic examination.<br>4. Patient has at least 2 of the following signs or symptoms at the affected site with no other recognized cause: localized pain or tenderness, redness, swelling, or heat<br>And at least 1 of the following:<br>1. organisms cultured from blood<br>2. positive antigen test performed on blood or urine (eg, H influenzae, S pneumoniae, N meningitidis, Group B Streptococcus, Candida spp)<br>3. diagnostic single antibody titer (IgM) or 4-fold increase in paired sera (IgG) for pathogen.
**Wound dehiscence**	Any dehiscence of the fascia>3 cm.

© 2022 American Medical Association. All rights reserved.

**Bleeding**	Necessary blood transfusion.
**Anastomotic leak**	Any dehiscence with clinical and radiological evidence.
**Respiratory failure**	Presence of dyspnea and respiratory rate>35/min or PaO2<60 mmHg or PaCO2>50 mmHg.
**Circulatory insufficiency**	Unstable blood pressure requiring use of extra fluids and/or cardiac stimulants.
**Renal failure**	Necessary hemodialysis.
**Renal dysfunction**	Defined as creatinine 200% above normal range.
**Hepatic dysfunction**	Defined as alanine or aspartate transaminase/bilirubin 200% above normal range or increased serum bilirubin concentration (50% above baseline).
**Pancreatic fistula**	Daily output of fluid >10 mL from surgical drainage with amylase concentration five times higher than that in serum.
**Delayed gastric emptying**	Necessity of nasogastric suction for more emptying than 8 days after surgery.
**MODS**	A state of physiological derangement in which organ function is not capable of maintaining homoeostasis.

Abbreviations: WBC, white blood cell count; **PaO2,** arterial partial pressure of oxygen; **MODS,** multiple organ dysfunction syndrome.

© 2022 American Medical Association. All rights reserved.

eTable 4. Types and characteristics of surgical procedures

	E-SPN Group (n=115)	L-SPN Group (n=114)	P Value
**The operation type—no. (%)**			.356
Laparotomy	60 (52.2)	52 (45.6)	
laparoscopic surgery	55 (47.8)	62 (54.4)	
**Surgical procedure—no. (%)**			.275
Gastrectomy (total/subtotal)	50 (43.5)	40 (35.1)	
Colectomy/Rectectomy	40 (34.8)	46 (40.4)	
PD/PPPD	13 (11.3)	19 (16.7)	
Major hepatectomy	1 (0.9)	3 (2.6)	
Others	11 (9.6)	6 (5.3)	
**Intraoperative contamination**			.224
None	108 (93.9)	104 (91.2)	
Mild	4 (3.5)	9 (7.9)	
Severe	3 (2.6)	1 (0.9)	
**Duration of operative time**			.636
<2 h	10 (8.7)	9 (7.9)	
2–5 h	85 (73.9)	90 (78.9)	
>5 h	20 (17.4)	15 (13.2)	
**Blood loss—no. (%)**			.362
Unknown	11 (9.6)	18 (15.8)	
≤500 mL	88 (76.5)	82 (71.9)	
>500 mL	16 (13.9)	14 (12.3)	
**Blood transfusion—no. (%)**	17 (14.8)	13 (11.4)	.558

Data are the numbers of participants (%). Outcomes expressed as percentages of patients with each outcome were compared between the two groups using the $\chi 2$ or Fisher's exact test.

Abbreviations: no., number; **E-SPN**, early supplemental parenteral nutrition; **L-SPN**, late supplemental parenteral nutrition; **PPPD**, pylorus-preserving pancreaticoduodenectomy; **PD**, pancreaticoduodenectomy; **Others**, gastroenterostomy, and pancreectomy.

© 2022 American Medical Association. All rights reserved.

eTable 5. Mean energy and protein delivery during days 3-12 in the intervention

	E-SPN Group (n=115)	L-SPN Group (n=114)	P Value
**During day 3-7 in the intervention**			
Received energy from EN (kcal/day)	703±245	684±233	.170
Received energy from PN (kcal/day)	1018±285	177±48	.000
Received energy from total nutrition support (kcal/day)	1720±368	861±254	.000
Received energy from total nutrition support (kcal/kg/day)	26.5±7.4	15.1±4.8	.000
Received protein from EN (g/day)	28.2±9.9	27.4±9.3	.162
Received protein from PN (g/day)	38.2±10.7	NA	
Received protein from total nutrition support (g/day)	66.4±14.1	27.4±9.3	.000
Received protein from total nutrition support (g/kg/day)	1.02±0.28	0.48±0.17	.000
**During day 8-12 in the intervention**			
Received energy from EN (kcal/day)	1282±239	1219±256	.001
Received energy from PN (kcal/day)	558±167	523±173	.010
Received energy from total nutrition support (kcal/day)	1839±294	1741±342	.000
Received energy from total nutrition support (kcal/kg/day)	28.8±6.2	29.6±7.2	.168
Received protein from EN (g/day)	51.3±9.6	48.7±10.3	.001
Received protein from PN (g/day)	20.9±6.2	19.5±6.5	.008
Received protein from total nutrition support (g/day)	72.2±11.5	68.3±13.4	.000
Received protein from total nutrition support (g/kg/day)	1.17±0.25	1.20±0.28	.346

Data are mean (SD) unless otherwise noted. Continuous data described as mean (SD) were compared using the t test or Mann–Whitney U test. Daily energy and protein intake were recorded from day 3 to day 12 after surgery.

Abbreviations: E-SPN, early supplemental parenteral nutrition; **L-SPN**, late supplemental parenteral nutrition; **NA**, not applicable.

© 2022 American Medical Association. All rights reserved.

eTable 6. Distribution of non-infectious complications during the intervention and follow-up

	E-SPN Group (n=115)	L-SPN Group (n=114)	Risk Difference (95% CI)	P Value
**Major non-infectious—no. (%)**	14 (12.2)	19 (16.7)	4.5 (-4.6 to13.6)	.353
Anastomotic leak	2 (1.7)	2 (1.8)		
Wound dehiscence	2 (1.7)	2 (1.8)		
Bleeding	2 (1.7)	2 (1.8)		
Intestinal obstruction	2 (1.7)	3 (2.6)		
Hemoperitoneum	1 (0.9)	0 (0.0)		
Arrythmia	0 (0.0)	1 (0.9)		
Hepatic dysfunction	1 (0.9)	1 (0.9)		
Renal dysfunction	1 (0.9)	1 (0.9)		
Respiratory failure	4 (3.5)	7 (6.1)		
**Minor non-infectious—no. (%)**	17 (14.8)	19 (16.7)	1.9 (-7.5 to 11.3)	.720
Pleural effusion	14 (12.2)	17 (14.9)		
Atelectasis	3 (2.6)	2 (1.8)		
**Clavien-Dindo classification**				
Grade I-II	22 (19.1)	25 (21.9)	2.8 (-7.7 to 13.3)	.627
Grade III-IV	9 (7.8)	13 (11.4)	3.6 (-4.0 to 11.2)	.380
**Total non-infectious—no. (%)**				
No. of patients affected (%)	31 (27.0)	38 (33.3)	6.4 (-5.5 to 18.2)	.316

Data are number of participants (%). Outcomes expressed as percentages of patients with each outcome were compared between the two groups using the Fisher's exact test.
Abbreviations: E-SPN, early supplemental parenteral nutrition; **L-SPN**, late supplemental parenteral nutrition.

© 2022 American Medical Association. All rights reserved.

eTable 7. Distribution of gastrointestinal intolerance complications and parenteral nutrition-related complications during the intervention and follow-up

	E-SPN Group (n=115)	L-SPN Group (n=114)	Risk Difference (95% CI)	P Value
Vomiting	9 (7.8)	15 (13.2)	5.3 (-2.6 to 13.2)	.203
Abdominal distension	57 (49.6)	64 (56.1)	6.6 (-6.3 to 19.5)	.355
Abdominal pain	28 (24.3)	18 (15.8)	-8.6 (-18.9 to 1.8)	.137
Diarrhea	16 (13.9)	19 (16.7)	2.8 (-6.6 to 12.1)	.587
Constipation	5 (4.3)	5 (4.4)	0.04 (-5.3 to 5.3)	.989
**Total GI intolerance complications—no. (%)**	67 (58.3)	79 (69.3)	11.0 (-1.3 to 23.4)	.099
Hyperglycemia/hypoglycemia	7 (6.1)	3 (2.6)	-3.5 (-8.7 to 1.8)	.333
Hyperlipidemia	2 (1.7)	1 (0.9)	-0.9 (-3.8 to 2.1)	.566
**PN-related complications—no. (%)**	9 (7.8)	4 (3.5)	-4.3 (-10.3 to 1.6)	.253
**Total number of patients affected—no. (%)**	75 (65.2)	82 (71.9)	6.7 (-5.3 to 18.7)	.320

Data are the numbers of participants (%). Outcomes expressed as percentages of patients with each outcome were compared between the two groups using the $\chi 2$ or Fisher's exact test.

Abbreviations: E-SPN, early supplemental parenteral nutrition; **L-SPN**, late supplemental parenteral nutrition; **GI**, gastrointestinal.

Parenteral nutrition-related complications: hyperglycemia, hypoglycemia, hyperlipidemia.

© 2022 American Medical Association. All rights reserved.

eTable 8. Hematological parameters at the end of the intervention

	E-SPN Group (n=115)	L-SPN Group (n=114)	Difference (95% CI)	P Value
**Nutritional indicators**				
Albumin (g/L)	35.5±7.6	33.7±4.5	-1.9 (-3.5 to -0.3)	.020
Prealbumin (mg/L)	158.4±38.1	130.0±36.3	-28.5 (-38.2 to -18.8)	.000
Transferrin (g/L), median (IQR)	1.8 (1.4-2.3)	1.8 (1.4-2.2)	-0.13 (-0.32 to 0.05)	.957
Retinol binding protein (mg/L)	24.8±12.2	22.4±12.3	-0.80 (-3.33 to 1.74)	.153
**Hepatic and renal function**				
ALT (U/L), median (IQR)	23.0 (13.0-51.0)	28.0 (16.0-52.0)	11.5 (-14.7 to 37.6)	.390
AST (U/L), median (IQR)	28.0 (19.0-50.0)	29.8 (19.0-55.0)	11.4 (-11.5 to 34.3)	.329
ALP (U/L), median (IQR)	62.0 (51.2-76.0)	64.0 (53.0-85.0)	5.9 (-15.7 to 27.4)	.594
TBiL (μmol/L), median (IQR)	14.2 (9.7-19.1)	14.4 (10.3-18.6)	-4.2 (-13.6 to 5.3)	.384
Blood urea nitrogen (mmol/L)	5.7±2.4	5.5±2.2	-0.22 (-0.82 to 0.36)	.271
Serum Creatinine (μmol/L)	71.8±26.8	72.0±21.7	0.2 (-6.2 to 6.5)	.954
**Metabolism-related index**				
Blood glucose (mmol/L)	6.6±3.8	7.3±3.4	0.72 (-0.22 to1.66)	.135
Total cholesterol (mmol/L)	2.96±1.31	3.15±1.21	0.19 (0.05 to 0.52)	.252
Triglyceride (mmol/L)	0.9±0.6	0.8±0.5	-0.1 (-0.26 to 0.02)	.099
HDL (mmol/L), median (IQR)	1.00 (0.90-1.21)	1.14 (0.92-1.44)	0.12 (0.04 to 0.20)	.372
LDL (mmol/L)	2.22±0.89	2.27±0.88	0.05 (-0.18 to 0.28)	.685
**Inflammatory biomarkers**				
White blood cell	7.26±2.74	7.82±2.56	0.56 (-0.24 to 1.57)	.113
C-reactive protein, median (IQR)	19.1 (11.3-35.6)	23.5 (12.3-37.8)	2.3 (-4.2 to 8.9)	.483

Data are mean (SD) unless otherwise noted. Continuous data described as mean (SD) were compared using the t test or Mann–Whitney U test.

Abbreviations: E-SPN, early supplemental parenteral nutrition; **L-SPN**, late supplemental parenteral nutrition; **ALT**, Alanine aminotransferase; **AST**, Aspartate aminotransferase; **ALP**, alkaline phosphatase; **TBiL**, total bilirubin; **HDL**, high-density lipoprotein; **LDL**, Low-density lipoprotein.

© 2022 American Medical Association. All rights reserved.

eFigure 1. Trial design

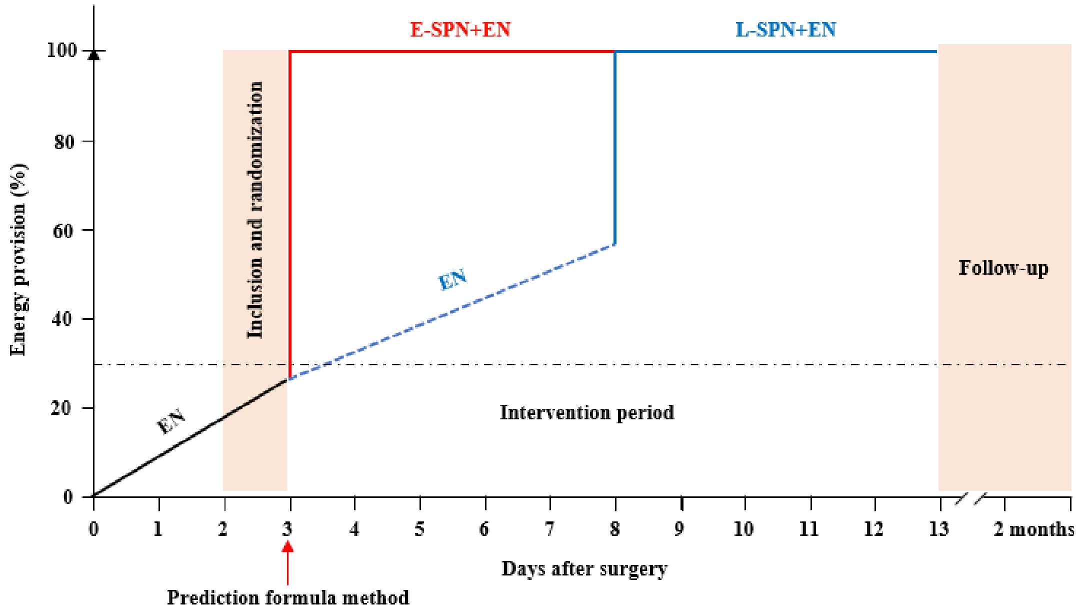

The black solid line shows the potential progression of EN in all patients before inclusion into the trial (Day 2), and the red line shows the energy delivery in patients on EN with E-SPN during the intervention period (Days 3-12), resulting in the potential prescription of 100% of the energy target (determined by prediction formula, 2 days after surgery). The blue broken line shows the potential energy provision for patients remaining on EN only (Days 3-7), and the blue solid line the energy delivery in patients on EN with L-SPN during the intervention period (Days 8-12), resulting in the potential prescription of 100% of the energy target.

EN=enteral nutrition, **E-SPN**=early supplemental parenteral nutrition, **L-SPN**=late supplemental parenteral nutrition.

© 2022 American Medical Association. All rights reserved.

eFigure 2. Kaplan-Meier analysis of nosocomial infections

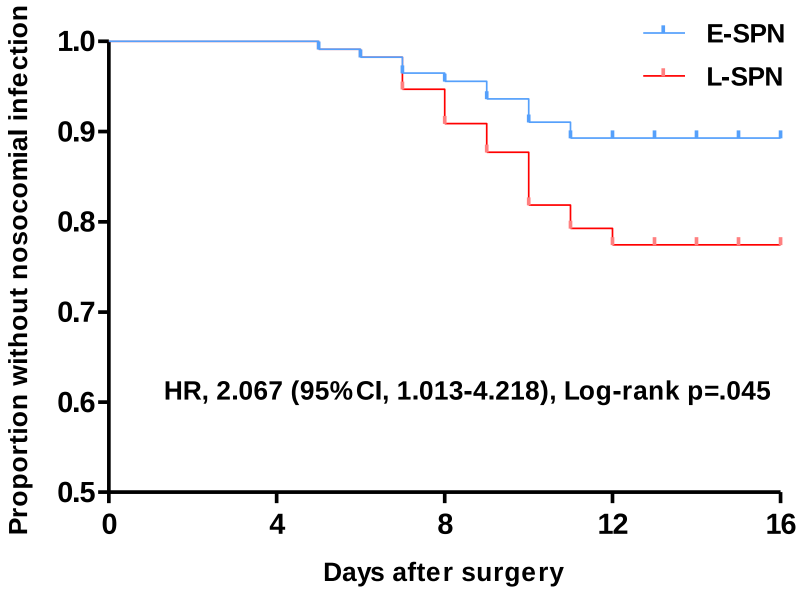

No. at risk					
E-SPN Group	115	115	105	33	6
L-SPN Group	114	114	99	43	9

E-SPN, early supplemental parenteral nutrition; **L-SPN**, late supplemental parenteral nutrition.

The patients were monitored for postoperative complications by two experienced physicians not associated with the surgical teams. They classified patients as "with or without infections" on the basis of clinical symptoms and laboratory examinations. Complications were classified by objective criteria as major or minor, and as infectious or noninfectious (**eTable 1**) according to a previously described classification[5, 6].

eReferences

1. Horan TC, Andrus M, Dudeck MA. **CDC/NHSN surveillance definition of health care-associated infection and criteria for specific types of infections in the acute care setting**. *American journal of infection control* 2008; 36(5):309-332.

2. Levy MM, Fink MP, Marshall JC, Abraham E, Angus D, Cook D, et al. **2001 SCCM/ESICM/ACCP/ATS/SIS International Sepsis Definitions Conference**. *Crit Care Med* 2003; 31(4):1250-1256.

3. Futier E, Garot M, Godet T, Biais M, Verzilli D, Ouattara A, et al. **Effect of Hydroxyethyl Starch vs Saline for Volume Replacement Therapy on Death or Postoperative Complications Among High-Risk Patients Undergoing Major Abdominal Surgery: The FLASH Randomized Clinical Trial**. *Jama* 2020; 323(3):225-236.

4. Spalding MC, Cripps MW, Minshall CT. **Ventilator-Associated Pneumonia: New Definitions**. *Critical care clinics* 2017; 33(2):277-292.

5. Bozzetti F, Braga M, Gianotti L, Gavazzi C, Mariani L. **Postoperative enteral versus parenteral**

© 2022 American Medical Association. All rights reserved.

nutrition in malnourished patients with gastrointestinal cancer: a randomised multicentre trial. *Lancet (London, England)* 2001; 358(9292):1487-1492.
6. Pacelli F, Bossola M, Papa V, Malerba M, Modesti C, Sgadari A, et al. **Enteral vs parenteral nutrition after major abdominal surgery: an even match**. *Archives of surgery (Chicago, Ill : 1960)* 2001; 136(8):933-936.

© 2022 American Medical Association. All rights reserved.
