# Supplementary material for: Effect of Early vs Late Supplemental Parenteral Nutrition in Patients Undergoing Abdominal Surgery: A Randomized Clinical Trial
Source: JAMA Surg. 2022 Mar 16;157(5):384–93. doi: 10.1001/jamasurg.2022.0269 (PMC8928091; doi:10.1001/jamasurg.2022.0269)
Supplement: Supplement 3. — Data Sharing Statement [file jamasurg-e220269-s003.pdf]

Data Sharing Statement

Data
Data available: Yes
Data types: Deidentified participant data
How to access data: wangxinying@nju.edu.cn
When available: With publication

Supporting Documents
Document types: Statistical/analytic code, Informed consent form
How to access documents: The request for the analytic code shall be sent to Prof. Xinying Wang, Email-address: wangxinying@nju.edu.cn
When available: With publication

Additional Information
Who can access the data: Data will be made available to researchers who provide a methodologically sound proposal to achieve aims in the approved proposal
Types of analyses: Specified purposes only
Mechanisms of data availability: Signed data access agreement
